# Spatial Engineering Direct Cooperativity between Binding Sites for Uranium Sequestration

**DOI:** 10.1002/advs.202001573

**Published:** 2020-12-04

**Authors:** Qi Sun, Yanpei Song, Briana Aguila, Aleksandr S. Ivanov, Vyacheslav S. Bryantsev, Shengqian Ma

**Affiliations:** ^1^ Department of Chemistry University of South Florida 4202 E. Fowler Avenue Tampa FL 33620 USA; ^2^ Department of Chemistry University of North Texas 1508 W Mulberry St Denton TX 76201 USA; ^3^ Chemical Sciences Division Oak Ridge National Laboratory P. O. Box 2008 Oak Ridge TN 37831 USA

**Keywords:** cooperative binding, environmental remediation, porous organic frameworks, radionuclide sequestration, uranium recovery

## Abstract

Preorganization is a basic design principle used by nature that allows for synergistic pathways to be expressed. Herein, a full account of the conceptual and experimental development from randomly distributed functionalities to a convergent arrangement that facilitates cooperative binding is given, thus conferring exceptional affinity toward the analyte of interest. The resulting material with chelating groups populated adjacently in a spatially locked manner displays up to two orders of magnitude improvement compared to a random and isolated manner using uranium sequestration as a model application. This adsorbent shows exceptional extraction efficiencies, capable of reducing the uranium concentration from 5 ppm to less than 1 ppb within 10 min, even though the system is permeated with high concentrations of competing ions. The efficiency is further supported by its ability to extract uranium from seawater with an uptake capability of 5.01 mg g^−1^, placing it among the highest‐capacity seawater uranium extraction materials described to date. The concept presented here uncovers a new paradigm in the design of efficient sorbent materials by manipulating the spatial distribution to amplify the cooperation of functions.

## Graphical abstract

The affinity of a specific chelating group in sorbent materials toward analyte of interest is significantly increased through engineering their spatial distribution, at an appropriate arrangement, driving the cooperative binding and thereby improved performance.

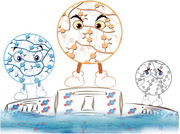

## 1 Introduction

With the decreasing availability of fossil fuels, to meet the ever‐growing energy demand, nuclear energy remains the most promising near‐term scalable replacement. Thus, acquiring the necessary fission fuels is a matter of energy security.^[^ ^1^ ^]^ Uranium is one such critical species, and the circumstance could be dramatically ameliorated if seawater were to be utilized as a source. In this case, the estimated uranium content amounts to four billion tons and exceeds terrestrial ores by nearly three orders of magnitude, which is enough to fuel the global nuclear power industry for centuries.^[^ ^2^ ^]^ However, the inadvertent release of radioactive materials into the environment poses potential severe threats to human health. Concurrently, with the closure of many nuclear and chemical weapons production facilities worldwide, an enormous legacy of uranium‐contaminated sites was left behind, also representing a major regional and national concern.^[^ ^3^ ^]^ In these contexts, technology development capable of sequestering uranium from seawater/wastewater in a highly cost‐effective manner would guarantee resource accessibility, assist in legacy waste site cleanup, and give a quick response to nuclear events. Given the complexity of these water samples, replete with enormous competing ions and a low concentration of uranium, it remains a tremendous challenge for achieving meaningful efficiencies. To fulfill these non‐trivial tasks, sorbent materials designed must possess an extremely high affinity toward uranium.^[^ ^4^, ^5^ ^]^

**Figure 1:**
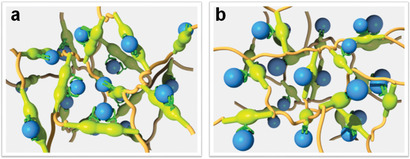
Schematic view of chelating site distributions. The functional groups in a convergent orientation allow cooperative binding to be triggered (a), while a non‐cooperative effect is seen with the functional groups randomly oriented (b).

Discoveries of the proper spatial arrangement of binding sites and cooperation between them to recognize specific metal ions with high sensitivity are two significant events in biology. It is perceived that instead of relying on a single strong binding, the use of cooperative chelating could be a propitious strategy for further improving the interactions (**Figure** **1**).^[^ ^6^ ^]^ Indeed, previous theoretical and crystallographic studies revealed that the uranyl ion bound with two ligands is more thermodynamically favorable.^[^ ^7^ ^]^ However, precise tuning of these aspects is largely missing in the design of synthetic adsorbents. Whereby, an intense focus has been placed on the synthesis of functional scaffolds by anchoring different chelating groups or the use of various supports. Given the fact that the binding sites are fixed on the sorbent materials, such synergies are only feasible if the correct distribution authorizes a cooperative binding mechanism. We therefore envisaged that the affinity of a specific chelating group toward uranium could be engineered by their spatial distribution in the adsorbents, at an appropriate arrangement, allowing for the occurrence of cooperative binding and thereby improved affinity.

To apply these considerations to specific examples, we sought to design uranium‐specific binding adsorbents aiming at establishing correlations between the spatial distribution of chelating groups and the property of the resulting adsorbents. To implement this study, we chose phosphorylurea as a chelating group due to its high affinity toward uranium.^[^ ^8^ ^]^ Regarding the scaffolds, porous organic polymers (POPs) were utilized for regulating the location of the phosphorylurea ligands relative to each other to understand how synergistic pairings can amplify the binding affinity. This choice is due to the level of synthetic control of POPs, which allows one to manipulate the compositions and local environments with high fidelity.^[^ ^9^ ^]^ Sorption tests revealed that the adsorbent with the chelating groups in a convergent orientation, magnifying synergistic effects, displayed an extraordinary affinity and selectivity for uranyl, far outperforming those in a random distribution. The great potential of this adsorbent is demonstrated by its excellent performance as a uranium scavenger with real‐world water samples, including the naturally occurring uranium in seawater. Moreover, the densely populated chelating groups on the porous framework allow ready access to binding sites and, thus, enable high uptake capacities and fast kinetics in uranium extraction.

## 2 Result and Discussion

**Scheme 1:**
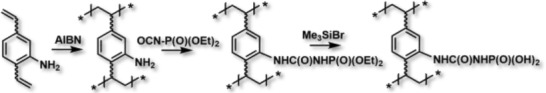
Synthetic route of phosphorylurea functionalized polymers.

The phosphorylurea functionalized porous polymers were synthesized following a combination of a de novo and post‐synthetic functionalization strategy—that is, first installing amine moieties on the monomers to have well‐defined anchoring sites and thereby the distributions of functionalities in the resulting materials, followed by carrying out a stepwise post‐synthetic modification. Accordingly, we initially constructed various amine monomers into highly porous frameworks. For this purpose, the amine moieties (aniline, 2,2′‐biphenyldiamine, and [1,1′:4′,1″‐terphenyl]‐2′‐amine) were equipped with a polymerizable vinyl group. The polymerization of these monomers was conducted in dimethylformamide (DMF) at 100 °C in the presence of a free radical initiator, azobisisobutyronitrile (AIBN), with the resulting polymers, denoted as POP1‐NH_2_, POP2‐NH_2_, and POP3‐NH_2_, respectively. The conversion of the amino polymers into the phosphorylurea‐derived polymers was accomplished through the addition reaction between the amine and diethoxyphosphinyl isocyanate (POP‐PO(OEt)_2_), followed by hydrolysis using Me_3_SiBr (POP‐PO_3_H_2_, see **Scheme** **1**).^[^ ^8^ ^]^ Here, the polymer constructed by biphenyldiamine (POP2‐NH_2_) and the corresponding materials from subsequent post‐synthetic grafting are chosen as representative samples for thorough descriptions.

The successful formation of the porous polymers with diethoxyphosphorylurea functionalities, and the subsequent hydrolysis was confirmed by Fourier transform infrared (FT‐IR) spectroscopy, elemental analysis, and solid‐state nuclear magnetic resonance (NMR) spectroscopy. The appearance of characteristic C=O and P—O—C bands at 1700 and 960–1050 cm^−1^, respectively, indicated the installation of diethoxyphosphorylurea in POP2‐NH_2_.^[^ ^10^ ^]^ Two primary amine N—H stretching bands at 3440 and 3355 cm^−1^ in the FT‐IR spectrum of POP2‐NH_2_ turned into one after being treated with OCN‐P(O)(OEt)_2_, suggestive of the high efficiency of this conversion (Figure S1, Supporting Information). To quantify the degree of the post‐synthetic modification, elemental analysis was used to determine the content of P species in POP2‐PO(OEt)_2_. The results showed that the weight percentage of P species in POP2‐PO(OEt)_2_ was 10.1 wt%, corresponding to around 97% of the amine groups involved in the condensation reaction and thus confirming high throughput of this transformation. Hydrolysis of POP2‐PO(OEt)_2_ to POP2‐PO_3_H_2_ led to a shift of the P=O band to 1052 cm^−1^, together with the appearance of P—O—H bands at 946 cm^−1^ in the FT‐IR spectra. The absence of P—O—C bands indicated the complete hydrolysis of ester groups. Further, in contrast with the solid‐state ^13^C NMR spectrum of POP2‐NH_2_, distinct ^13^C chemical shifts were monitored in the final POP2‐PO_3_H_2_ product. Also, the signature peak at 152.2 ppm for the C=O group in POP2‐PO_3_H_2_ confirmed the successful incorporation of dihydroxyphosphorylurea groups (Figure S2, Supporting Information). Moreover, data from solid‐state ^31^P NMR experiments of POP2‐PO_3_H_2_ gave an intense singlet peak at −5.4 ppm, characteristic of —PO_3_H_2_, thus providing additional evidence for the success of post‐synthetic modification (Figure S2, Supporting Information). The scanning electron microscopy and transmission electron microscopy results suggested that the morphology of the polymer before and after modification were maintained, both of them appearing as interconnected meso‐ and macroporous ensembles composed of randomly agglomerated small particles (Figures S3 and S4, Supporting Information). Nitrogen sorption isotherms collected at 77 K revealed that POP2‐PO_3_H_2_ retained permanent porosity, giving a relatively high BET surface area of 371 m^2^ g^−1^, albeit smaller than the surface area of parent POP2‐NH_2_ (687 m^2^ g^−1^, Figure S5, Supporting Information). Both POP2‐NH_2_ and POP2‐PO_3_H_2_ exhibit similar sorption behavior of type I plus type IV, validating their hierarchical porous structures comprised of both micropores and mesopores. Altogether, the results above indicate that the phosphorylurea ligands have been densely anchored on the porous framework. The detailed characterizations of other materials are shown in the Supporting Information (**Table** **1** and Figures S6–S15, Supporting Information).

**Table 1:** Cartoon structures of various phosphorylurea functionalized hierarchical porous polymers and their corresponding textural parameters

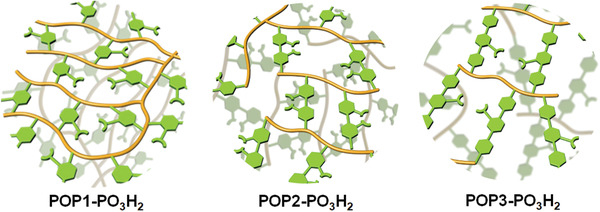			
POP	Structure^a)^	BET surface area [m^2^ g^−1^]	Pore volume [cm^3^ g^−1^]
POP1‐PO_3_H_2_	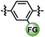	412	0.45
POP2‐PO_3_H_2_	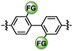	371	0.43
POP3‐PO_3_H_2_	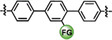	571	0.54

^a)^ FG = –NHC(O)NHP(O)(OH)_2_.

With these adsorbents in hand, we investigated their binding with uranium species using various spectroscopic techniques, including elemental distribution mapping via energy‐dispersive X‐ray spectroscopy (EDX), FT‐IR spectroscopy, X‐ray absorption fine structure (XAFS) spectroscopy, and X‐ray photoelectron spectroscopy (XPS). The EDX elemental mapping for the uranyl reacted samples (U@POP‐PO_3_H_2_), which were obtained by treating POP‐PO_3_H_2_ with the uranium aqueous solution (20 ppm, 400 mL, pH ≈ 5) overnight, revealed the presence of significant amounts of uranium species (Figure S16, Supporting Information). Further evidence for the incorporation of uranium species came from a strong vibration of [O=U=O]^2+^ in the FT‐IR spectra of U@POP‐PO_3_H_2_ (Figure S17, Supporting Information). The EXAFS spectra of the U loaded samples show a prominent FT peak at ≈3.0 Å attributable to the uranium–phosphate interaction, indicative of the occurrence of the coordination between the phosphorylurea functionality and uranyl (Figure S18, Supporting Information).^[^ ^8b^ ^]^ In the XPS spectra, the U4*f*_5/2_ binding energies appeared at 393.9, 393.8, and 394.5 eV for U@POP1‐PO_3_H_2_, U@POP2‐PO_3_H_2_, and U@POP3‐PO_3_H_2_, respectively, significantly lower than that of UO_2_(NO_3_)_2_∙6H_2_O (393.4 eV), indicative of the coordination between the phosphorylurea groups and uranium species (Figures S19–S22, Supporting Information). Whereas, the differences in the binding energy of the uranium species among these three adsorbents suggest coordination dissimilarities, which could lead to disparate sorption performance.

**Figure 2:**
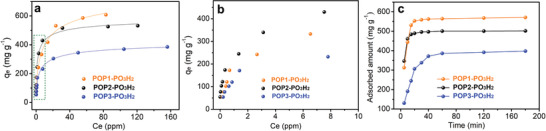
Uranium adsorption isotherms and kinetics investigations. a) Uranium sorption isotherms for POP based adsorbents. Conditions: 5 mg of each material was added into 10 mL aqueous solutions with different uranium concentrations and stirred vigorously overnight. The lines are fitted with the Langmuir model; all the fits have *R* ^2^ values higher than 0.99. b) Enlarged section of the green rectangle in (a). c) The kinetics of uranium adsorption from aqueous solutions with an initial concentration of 20 ppm (400 mL), at pH ≈ 5, and adsorbent material (5 mg).

Given this, we started to evaluate their overall capacities for uranium from the fitting of adsorption isotherms collected after equilibrating the sorbent materials with aqueous uranium solutions with the concentrations ranging from 27 to 387 ppm (Figure 2a). The best fit to these experimental data utilized a Langmuir model with the correlation coefficient values (*R* ^2^) higher than 0.99 (Figure 2b and Figure S23, Supporting Information). Notably, for the low concentrations most relevant to uranium captured from real‐world water samples, POP2‐PO_3_H_2_ displayed much steeper adsorption at low uranium concentrations than both POP1‐PO_3_H_2_ and POP3‐PO_3_H_2_, indicative of stronger binding sites. This enhanced uptake notably persisted over an entire range of uranium concentrations tested relative to POP3‐PO_3_H_2_, whereas it was overridden by POP1‐PO_3_H_2_ at high concentrations. Considering the relatively high surface areas and the swellability of these sorbent materials in water, it is assumed that the chelators therein can be fully accessible. Therefore, the uptake capacities of the adsorbents should be primarily determined by the content of functional groups. However, as revealed by elemental analysis, the dihydroxyphosphorylurea group content in these adsorbents was in the order of POP2‐PO_3_H_2_ > POP1‐PO_3_H_2_ > POP3‐PO_3_H_2_, contradicting the experimental uptake capacities reflected by their adsorption isotherms. To explain this, we presumed that the uranium species might have different coordination fashions in these adsorbents. In POP2‐PO_3_H_2_, two chelating sites are adjacent to each other, driving the formation of the complex with two phosphorylurea ligands that bind to one uranyl ion. On the other hand, in POP1‐PO_3_H_2_ and POP3‐PO_3_H_2_, part of the functionalities cannot participate in cooperative binding due to the considerable strain of polymer chains in a highly cross‐linked polymer that exists one to one with the uranyl ion and, thus, results in a higher uptake capacity. To rationalize these assumptions, we compared the experimental saturation capacities with the theoretical values of these materials by evaluating their uptake capacities from a 20 ppm uranium aqueous solution to minimize the physical adsorption at a phase ratio (V/m) of 80 000 mL g^1^. After reaching equilibrium, the saturated sorption capacities were obtained under the experimental conditions as 571, 502, and 398 mg g^−1^ for POP1‐PO_3_H_2_, POP2‐PO_3_H_2_, and POP3‐PO_3_H_2_, respectively (Figure 2c, average of three batches with the differences of each being in the range of ±3%). Accordingly, the coordination numbers of the ligand to a uranyl ion were calculated to be 1.55, 1.86, and 1.31, respectively, consistent with our hypothesis (Table S1, Supporting Information).

To shed more light on the binding, we contrasted their affinity toward uranium, which can be expressed in terms of the distribution coefficient (*K*_d_, for definition, see the Experimental Section). With the knowledge that the uranyl complex involved with two dihydroxyphosphorylurea groups exhibits higher thermodynamic stability in comparison with one to one, the *K*_d_ values were therefore measured in the presence of two equivalents of immobilized ligands against one equivalent of uranyl in the corresponding amount of aqueous solutions. After reaching equilibrium POP2‐PO_3_H_2_ extracted 99.98% of uranium from solution corresponding to a decrease in uranium concentration from 10 ppm to 1.8 ppb, whereas the residual uranium concentrations treated by POP1‐PO_3_H_2_ and POP3‐PO_3_H_2_ were 23.7 and 179.7 ppb, respectively. In view of that, the *K*_d_ value for POP2‐PO_3_H_2_ was calculated to be as high as 2.5 × 10^8^ mL g^−1^ and this represents an affinity which is more than one and two orders of magnitudes higher than that of POP1‐PO_3_H_2_ (1.8 × 10^7^ mL g^−1^) and POP3‐PO_3_H_2_ (1.5 × 10^6^ mL g^−1^), respectively. Considering the same amount of chelating sites used, their discrepancy in uranium removal efficiencies confirmed the role of ligand distribution in the adsorbents. With these pieces of information, we can now formulate clear design rules to achieve high‐performance sorbent materials, increasing the density of functional groups facilitates their cooperative binding, which could be further improved after being oriented convergently.

**Figure 3:**
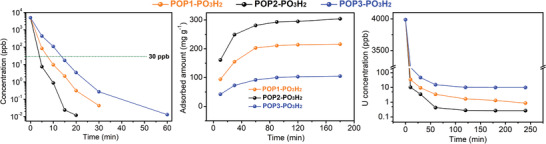
Uranium sorption from potable water and simulated seawater samples. a) The kinetics of uranium removal efficiency for various adsorbents from potable water spiked with uranium (5000 ppb) at V:m = 40 000 mL g^−1^. b) The kinetics of uranium adsorption for various adsorbents in simulated seawater solutions with an initial uranium concentration of 10.3 ppm at V:m = 13 000 mL g^−1^. c) The kinetics of uranium removal efficiency from simulated seawater spiked with uranyl (4056 ppb) at V:m = 2000 mL g^−1^.

To validate these results, we evaluated their efficiency in the removal of uranium from real water samples, in which uranium species were intentionally spiked with a dilute concentration (around 5 ppm). Time‐course adsorption measurements revealed that uranium extraction by these adsorbents followed a pseudo‐second‐order model, suggesting a chemisorption mechanism with pseudo‐second‐order adsorption rate constants of 0.0724, 0.595, and 0.0182 mg mg^−1^ min^−1^, respectively (Figure S24, Supporting Information). POP2‐PO_3_H_2_ was especially kinetically efficient, reaching equilibrium capacity within 20 min and efficiently reduced the uranium content below the U.S. Environmental Protection Agency standard for drinking water of 30 ppb within 5 min (7.54 ppb). Under otherwise identical conditions, the residual uranium concentrations in the potable water samples were 85 and 437.4 ppb for POP1‐PO_3_H_2_ and POP3‐PO_3_H_2_, respectively, verifying the superior performance of POP2‐PO_3_H_2_ (**Figure** **3**a). Moreover, this material can be readily recycled by washing with Na_2_CO_3_ and HNO_3_ solutions with retained uranium removal efficiency and structural integrity for at least 5 cycles (Figure S25, Supporting Information). These preliminary uranium sorption results suggest these materials may find application for quick response to a nuclear event.

Subsequently, we investigated their potential applications in wastewater treatment. Compared to surface water samples, wastewater, such as stored nuclear tank wastes, is often comprised of significantly higher concentrations of competing ions. Given this, tests were carried out with a 5 ppm of uranium aqueous solution containing 100 ppm of various interfering ions, including radioactive ions (Cs^+^, Sr^2+^), lanthanides (Ce^3+^, La^3+^), and transition heavy metal ions (Zn^2+^, Co^2+^, Fe^3+^, Cu^2+^, Pb^2+^) as well as common ions (Na^+^, Mg^2+^, Ca^2+^) at pH ≈ 5. The tolerance of uranium uptake with these competitive ions present was very impressive. POP2‐PO_3_H_2_ was shown to remove more than 99.8% of uranium species from the synthetic wastewater, bringing uranium concentrations to 8.7 ppb, still within the drinkable regime. Moreover, the uranium extraction was kinetically favorable, reaching equilibrium within 20 min. These results imply that the removal efficiency is negligibly affected by these interferons, thereby showing its great potential in mitigating a critical problem in the long‐term storage of nuclear waste. In addition to UO_2_^2+^, part of lanthanides and transition heavy metal ions were also removed (Table S2, Supporting Information). More importantly, POP2‐PO_3_H_2_ is very robust, which can be fully recycled using 0.1 m of HNO_3_ as eluent. It should be noted that the removal efficiencies were reduced to 95.4% and 90.3% for POP1‐PO_3_H_2_ and POP3‐PO_3_H_2_, respectively, indicative of some competitive interference with these samples.

Armed with these exciting results that demonstrate POP2‐PO_3_H_2_ to be excellent for uranium adsorption, we next sought to apply it for extraction uranium from seawater. Since the ocean represents a virtually unlimited supply, this resource, if convincingly shown to be recoverable at a suitable coast, can establish a uranium price cap. To test the potential of the developed adsorbents for this application, we initially investigated their uranyl sequestration performance from synthetic seawater, which contains ≈10.3 ppm of uranium, 25.6 g L^−1^ of NaCl, and 0.198 g L^−1^ of NaHCO_3_. POP2‐PO_3_H_2_ exhibited an exceptional ability to capture uranium species with equilibrium reached within 300 min and an uptake capacity up to 304 mg g^−1^. Moreover, POP2‐PO_3_H_2_ was capable of reducing the uranium concentration to a shallow low level (≈1 ppb, removal capacities ≈99.9%, V:m = 2000 mL g^−1^). Together, these results demonstrate the feasibility of uranium mining from the ocean with this material. By contrast, under the otherwise identical conditions, POP1‐PO_3_H_2_ and POP3‐PO_3_H_2_ showed uptake capacities of 216 and 105 mg g^−1^, as well as residual uranium concentrations of around 10 and 25 ppb, respectively.

Encouraged by these results, efforts were then made to assess their adsorption ability of naturally occurring uranium species in seawater. The investigations were performed using adsorbents (5 mg) which were immersed separately in a tank containing 5 gallons of seawater and shaken at room temperature. Following 56‐days of contact, a seawater uranium recovery capacity of approximately 5.01 mg per gram of adsorbent was achieved for POP2‐PO_3_H_2_, superior to that afforded by POP1‐PO_3_H_2_ and POP3‐PO_3_H_2_ (3.82 and 1.13 mg U g^−1^, respectively), which also places it among the highest‐capacity seawater uranium extraction materials described to date (Table S3, Supporting Information).

**Figure 4:**
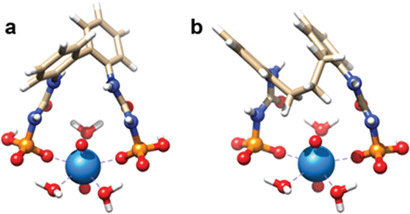
Structures of uranyl complexes depending on the linkage between the phosphorylurea functional groups. a) DFT optimized complex with two mono‐deprotonated functionalities linked through one C—C bond (“convergent orientation,” complex **1**). b) DFT optimized complex with two mono‐deprotonated functionalities linked through four C‐C bonds (“random orientation,” complex **2**). Color legend: P, orange; O, red; N, blue; C, beige; H, white; U, cyan.

Understanding the observed sorption efficiencies exhibited by phosphorylurea functional groups in the synthesized polymeric materials requires investigation of the uranyl binding with the sorbents at the molecular level. First‐principles calculations based on density functional theory (DFT) were performed for the representative polymer fragments to assess their binding affinity toward the uranyl (UO_2_^2+^) ion. Since polymer POP3‐PO_3_H_2_ shows the poorest efficiency, most likely due to the low density of functional groups, we focused our attention to study uranyl binding and structural characteristics for polymers POP1‐PO_3_H_2_ and POP2‐PO_3_H_2_, having more comparable uptake capacities. Speciation studies^[^ ^11^ ^]^ indicate that two functional groups are usually needed to bind one uranyl ion at higher ligand/uranyl concentration ratios, pointing to the importance of the relative orientation of the two phosphorylurea ligands in the polymers (as shown in **Figure** **4**). The question we ask here is how the intrinsic binding affinity of primary structural units of the polymers in the chelate coordination mode could affect the uranium adsorption performance. To address this, we performed calculations for a model of UO_2_^2+^ complexes with two mono‐deprotonated phenylcarbamoylphosphoramidic acids joined together through one (Figure 4a) and four (Figure 4b) C—C bonds, corresponding to the convergent and random orientation of the functional groups in POP2‐PO_3_H_2_ and POP1‐PO_3_H_2_, denoted as complex **1** and complex **2**, respectively. This computational model is justified, as the p*K*_a1_ of phosphorylurea can be approximated using the p*K*_a1_ = 2.38 of methylphosphonic acid,^[^ ^12^ ^]^ indicating that the functional groups on the synthesized polymers would already be in a mono‐deprotonated state at the experimental conditions.

To establish the most energetically stable structure of the respective uranyl complexes, DFT calculations were performed for various possible configurations (the lowest six uranyl complexes with their relative Gibbs free energies are shown in Figure S26, Supporting Information). While we considered both monodentate and bidentate coordination of uranyl by each phosphoryl group, only the monodentate binding motif was found to be stable, in agreement with the available crystal structures in the Cambridge Structural Database (CSD) showing exclusively monodentate phosphoryl binding for 1:1 uranyl complexes (Figure S27, Supporting Information). The results of our computational search reveal that monodentate uranyl binding with two phosphorylurea ligands forming a network of hydrogen bonds with the inner‐sphere water molecules is the most favored binding motif (Figure 4). This is consistent with the results of previous experimental^[^ ^13^ ^]^ and computational^[^ ^14^ ^]^ reports on similar ligands. Structural examinations of the uranyl complexes reveal slightly shorter bond lengths (2.284 and 2.307 Å) between uranium and phosphoryl oxygens for complex **1** compared to those (2.287 and 2.315 Å) for complex **2**; however, the opposite trend is observed for the inner‐shell water molecules, which are generally stronger bound to uranyl in complex **2** (Figure S28 and Table S4, Supporting Information). Natural bond orbital (NBO) analysis of ligand‐UO_2_^2+^ orbital interactions shows strong uranyl binding by both functionalities via dative *σ*‐bonds with the associated second‐order stabilization energies summarized in Table S5, Supporting Information.

In an effort to provide a thermodynamic assessment of uranyl complexation by the ligands in an aqueous medium, our theoretical approach^[^ ^11^ ^]^ based on quantum chemical calculations was applied, enabling us to estimate and compare the equilibrium constants (log *β*) for the formation of complexes in Figure 4. The results reveal that the two functionalities possess almost similar binding affinity toward UO_2_^2+^, with the ligand in complex **2** forming slightly stronger complexes (log *β* = 8.9) than the ligand in complex **1** (log *β* = 7.9). Therefore, we conclude that the thermodynamic calculations alone for the model complexes are not sufficient to rationalize the differences in the performance of the studied polymers. As the adsorption of uranium depends strongly on the accessibility and flexibility of active surface ligands, it is reasonable to assume that two phosphorylurea groups in polymer POP2‐PO_3_H_2_ can more easily reorient through rotations about a single C—C bond to adopt a suitable mode for uranyl complexation, while a similar transformation of randomly placed functional groups in polymer POP1‐PO_3_H_2_ would result in a higher energy penalty for structural reorganization. Indeed, the complex of 1:1 phosphorylurea group and uranyl gives a lower equilibrium constant value, with the most thermodynamically stable one of (log *β* = 6.4, Figures S29 and S30, Supporting Information). Overall, the results suggest that the additional constraint imposed by a polymer matrix play a more significant role than the intrinsic binding affinity of the constituent functional groups, effecting adsorbent performance.

## 3 Conclusion

The foregoing results established that pre‐organization of the receptors in adsorbents is a robust way for increasing the possibility of cooperative binding and thereby the superior affinity toward the target guests. This strategy that has been less studied before will be an important addition to toolboxes for adsorptive materials design. We experimentally demonstrated that POP2‐PO_3_H_2_, with dihydroxyphosphorylurea binding groups densely populated in an organized manner exhibited very high affinity toward uranium required for the treatment of fission products and the mining of energy materials from seawater. We anticipate that the straightforward and modular strategy presented here will provide a versatile tool for the rational engineering of sorbent materials to enable the efficient and specific encapsulation of target species.

## Conflict of Interest

The authors declare no conflict of interest.

## Supporting information

Supporting Information

## References

[1] a) M. S. Dresselhaus , I. L. Thomas , Nature 2001, 414, 332; 11713539 10.1038/35104599

[2] a) R. Taylor , Chem 2016, 1, 659;

[3] a) Q. Sun , B. Aguila , S. Ma , Trends Chem. 2019, 1, 292;

[4] a) D. S. Sholl , R. P. Lively , Nature 2016, 532, 435; 27121824 10.1038/532435a

[5] a) Q. Sun , B. Aguila , L. D. Earl , C. W. Abney , L. Wojtas , P. K. Thallapally , S. Ma , Adv. Mater. 2018, 30, 1705479; 10.1002/adma.201705479 29582484

[6] a) B. Chen , S. Xiang , G. Qian , Acc. Chem. Res. 2010, 43, 1115; 20450174 10.1021/ar100023y

[7] a) S. Vukovic , L. A. Watson , S. O. Kang , R. Custelcean , B. P. Hay , Inorg. Chem. 2012, 51, 3855; 22376298 10.1021/ic300062s

[8] a) M. Carboni , C. W. Abney , S. Liu , W. Lin , Chem. Sci. 2013, 4, 2396;

[9] a) A. G. Slater , A. I. Cooper , Science 2015, 348, aaa988;

[10] G. Barin , G. W. Peterson , V. Crocellà , J. Xu , K. A. Colwell , A. Nandy , J. A. Reimer , S. Bordiga , J. R. Long , Chem. Sci. 2017, 8, 4399. 30155218 10.1039/c6sc05079d PMC6100238

[11] A. P. Ladshaw , A. S. Ivanov , S. Das , V. S. Bryantsev , C. Tsouris , S. Yiacoumi , ACS Appl. Mater. Interfaces 2018, 10, 12580. 29580049 10.1021/acsami.7b17031

[12] P. C. Crofts , G. M. Kosolapoff , J. Am. Chem. Soc. 1953, 75, 3379.

[13] a) E. P. Horwitz , H. Diamond , K. A. Martin , Solvent Extr. Ion Exch. 1987, 5, 447;

[14] C.‐Z. Wang , J.‐H. Lan , Y.‐L. Zhao , Z.‐F. Chai , Y.‐Z. Wei , W.‐Q. Shi , Inorg. Chem. 2013, 52, 196. 23231505 10.1021/ic301592f

