# Supplementary material for: Spatial Engineering Direct Cooperativity between Binding Sites for Uranium Sequestration
Source: Adv Sci (Weinh). 2020 Dec 4;8(2):2001573. doi: 10.1002/advs.202001573 (PMC7816700; doi:10.1002/advs.202001573)
Supplement: Supplementary file 1 — Supporting Information [file ADVS-8-2001573-s001.pdf]

((Supporting Information can be included here using this template))

© 2020 Wiley-VCH GmbH

Supporting Information

for *Adv. Sci.,* DOI: 10.1002/advs.202001573

Spatial Engineering Direct Cooperativity between Binding Sites for Uranium Sequestration

*Q. Sun, Y. Song, B. Aguila, A. S. Ivanov, V. S. Bryantsev, and S. Ma**

© 2020 Wiley-VCH GmbH

Supporting Information

Spatial Engineering Direct Cooperativity between Binding Sites for Uranium Sequestration

*Q. Sun, Y. Song, B. Aguila, A. S. Ivanov, V. S. Bryantsev, and S. Ma**

Supporting Information

Spatial Engineering Direct Cooperativity between Binding Sites for Uranium Sequestration

*Q. Sun, Y. Song, B. Aguila, A. S. Ivanov, V. S. Bryantsev, and S. Ma**

Materials Synthesis

Synthesis of POP2-PO_3H_2

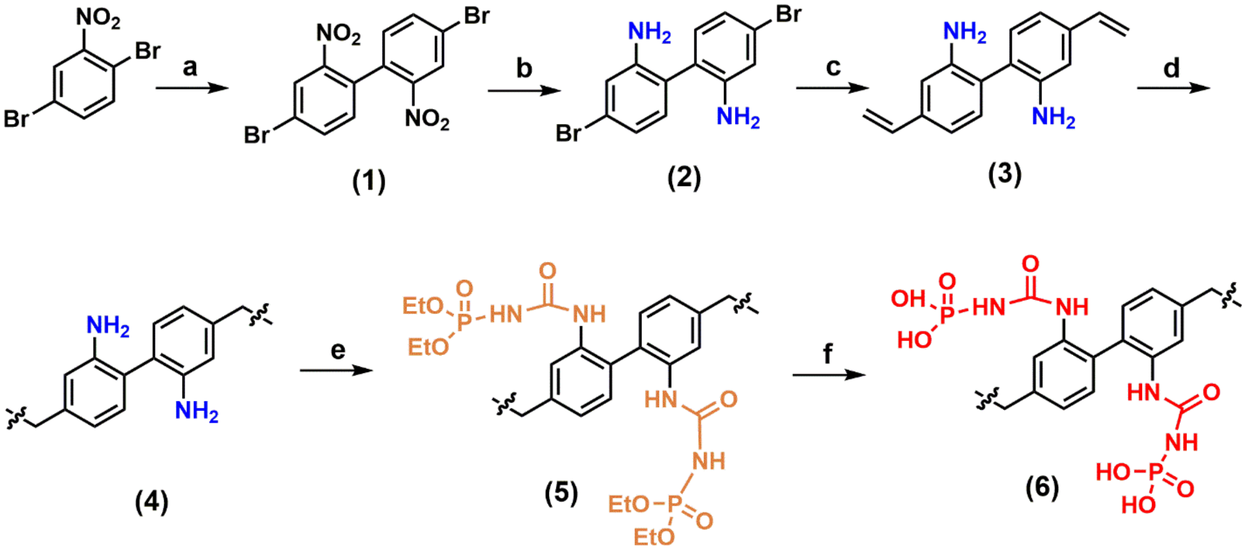

Reagents: (a) Cu; (b) Sn, concentrated HCl; (c) potassium vinyltrifluoroborate, $Pd(OAc)_2$; (d) AIBN, DMF; (e) OCN-P(O)$(OEt)_2$; (f) Me_3SiBr

4,4'-dibromo-2,2'-dinitrobiphenyl (1): Copper powder (6.0 g, 89.5 mmol) was added into a solution of 2,5-dibromonitrobenzene (14.0 g, 42,7 mmol) in dimethylformamide (DMF, 60 mL) and the resulting mixture was heated at 125 ºC for 3 h. After being cooled to room temperature, DMF was evaporated under high vacuum, followed by the introduction of toluene and removal of the insoluble materials by filtration. The filtrate was washed with 10% $NaHCO_3$, dried over Na_2SO_4, and evaporated, giving the crude product which was then purified by flash chromatography with hexane/ethyl acetate (2:1) as eluent to afford the title compound as a yellow solid. Yield: 6.2 g (72%). ^1H NMR (400 MHz, d_6-DMSO, 298 K, TMS): δ 8.41 (d, 2H, *J*=2 Hz), 8.03-8.06 (m, 2H), 7.45 (d, 2H, *J*=8.4 Hz) ppm.

***4,4'-dibromobiphenyl-2,2'-diamine (2)*: 1** (4.0 g, 10 mmol) and tin powder (5.0 g, 42 mmol) were dispersed in a mixture of ethanol (50 mL) and concentrated HCl (30 mL), and the resulting reaction mixture was then heated to reflux at 100 ºC for 2 h. After cooling, the mixture was poured into ice water (400 mL) and then made alkaline with 20 wt% NaOH aqueous solution. The product was extracted with diethyl ether, washed with brine, dried over Na_2SO_4, and evaporated to dryness to give the title compound as a light-brown solid that was used without further purification. Yield: 3.16 g (93%). ^1H NMR (400 MHz, d_6-DMSO, 298 K, TMS): δ 6.91 (s, 2H), 6.79 (d, 2H, *J*=8 Hz), 6.70-6.72 (m, 2H), 4.88 (s, 4H) ppm.

***4,4'-divinylbiphenyl-2,2'-diamine (3):* 2** (3.4 g, 10 mmol), potassium vinyltrifluoroborate (3.2 g, 24 mmol), K_2CO_3 (6.6 g, 48 mmol), PPh_3 (0.1 g, 0.4 mmol) and $Pd(OAc)_2$ (0.045 g, 0.2 mmol) were dissolved in a mixture of toluene (30 mL), THF (30 mL) and H_2O (6 mL), and the resulting mixture was refluxed at 90 °C under N_2 atmosphere for 24 h. The residue was extracted with

ethyl acetate, washed with brine, dried over Na_2SO_4, and evaporated under reduced pressure, giving the crude compound which was purified by flash chromatography with hexane/ethyl acetate (2:1) as eluent to afford the title compound as a yellow solid. Yield: 1.92 g (82%). ^{1}H NMR (400 MHz, $CDCl_3$, 298 K, TMS): δ 7.08 (d, 2H, *J*=6 Hz), 6.91 (s, 2H, *J*=6.4 Hz), 6.83 (s, 2H), 6.64-6.70 (m, 2H), 5.74 (d, 2H, *J*=14 Hz), 5.24 (d, 2H, *J*=8.4 Hz), 3.57 (s, 4H) ppm. ^{13}C NMR (125 MHz, $CDCl_3$, 298K, TMS) 144.11, 138.26, 136.70, 131.26, 124.18, 117.10, 113.86, 113.34 ppm.

Synthesis of POP2-PO$_3$H$_2$ (6): **3** (1.0 g) was dissolved in DMF (10 mL), followed by the addition of free radical initiator azobisisobutyronitrile (AIBN, 25 mg). After stirring at room temperature to achieve homogeneity, the mixture was transferred into a 20 mL autoclave and maintained at 100 °C for 24 h. A light yellow solid product **4** (1.0 g, 100% yield) was obtained by extracting the DMF solvent with EtOH and drying in vacuum at 50 °C for 12 h. Subsequently, 0.5 g of **4** was swollen in 20 mL of DMSO, followed by the addition of 0.91 g of OCN-P(O)$(OEt)_2$. After being stirred at 50 °C under N_2 atmosphere for 72 h, the mixture was filtered and washed with excess CH_2Cl_2, and finally dried at 50 °C under vacuum to yield **5** (1.21 g, 97%). Next, 0.5 g of **5** was suspended in 20 mL of CH_2Cl_2, followed by the addition of 2.5 mL of Me_3SiBr. After being stirred for 12 h, the material was isolated by filtration, washed with CH_2Cl_2, and then dispersed in the mixture of CH_2Cl_2 (20 mL) and water (5 mL). After being stirred at room temperature for 3 h, the mixture was filtered, washed with DMF, EtOH, and CH_2Cl_2 in sequence, and finally dried at 50 °C under vacuum to give the title product **6** (0.39 g, 96%).

Synthesis of POP1-PO$_3$H$_2$

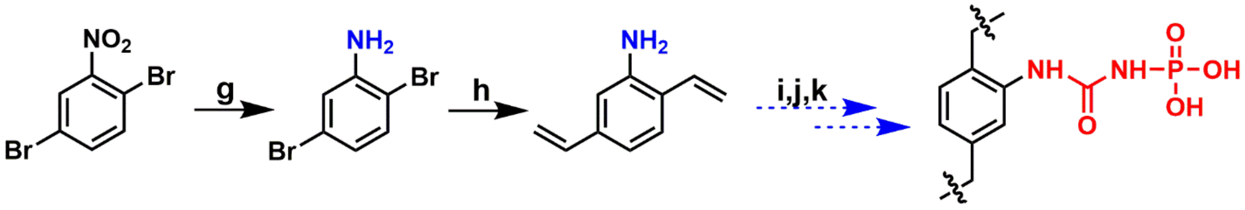

7 8 9

Reagents: (g) Sn, concentrated HCl; (h) potassium vinyltrifluoroborate, Pd(OAc)$_2$; (i,j,k) AIBN, DMF; OCN-P(O)(OEt)$_2$; Me$_3$SiBr

Synthesis of 2,5-dibromoaniline (7): 1,4-dibromo-2-nitrobenzene (2.5 g, 10 mmol) and tin powder (5.0 g, 42 mmol) were dispersed in a mixture of ethanol (50 mL) and concentrated HCl (30 mL), and the resulting mixture was then heated to reflux at 70 ºC for 12 h. After cooling, the mixture was poured into ice water (400 mL) and then made alkaline with 20 wt% NaOH aqueous solution. The product was extracted with diethyl ether, washed with brine, dried over Na_2SO_4, and evaporated to dryness, giving the crude compound which was purified by flash chromatography with hexane/ethyl acetate (5:1) as eluent to afford the title compound as white solid. Yield: 2.38 g (95%). ^{1}H NMR (400 MHz, d_6-DMSO, 298 K, TMS): δ 7.23 (d, 1H, *J*=8.4 Hz), 6.92 (d, 1H, 2.4 Hz), 6.55-6.58 (m, 1H), 5.55 (s, 2H) ppm.

Synthesis of 2,5-divinylaniline (8): **7** (2.5 g, 10 mmol), potassium vinyltrifluoroborate (3.3 g, 24 mmol), K_2CO_3 (5.52 g, 40 mmol), and $Pd(PPh_3)_4$ (275 mg, 0.25 mmol) were dissolved in a mixture of toluene (25 mL), THF (25 mL) and H_2O (5 mL), and the resulting mixture was refluxed at 90 °C under N_2 atmosphere for 36 h. The residue was extracted with ethyl acetate, washed with brine, dried over Na_2SO_4, and evaporated under reduced pressure, giving the crude compound which was purified by flash chromatography with hexane/ethyl acetate (5:1) as eluent to afford the title compound as a light brown solid. Yield: 1.17 g (81%). ^{1}H NMR (400 MHz, $CDCl_3$, 298 K, TMS): δ 7.25 (d, 1H, *J*=6.4 Hz), 6.82-684 (m, 1H), 6.57-6.76 (m, 1H), 6.70 (d, 1H, *J*=1.6 Hz), 5.60-5.70 (m, 2H), 5.18-5.30 (m, 2H), 3.73 (s, 2H) ppm. ^{13}C NMR (125 MHz, $CDCl_3$, 298K, TMS) 143.97, 138.28, 136.84, 132.53, 127.63, 123.92, 117.32 115.63, 114.02, 113.85 ppm.

Synthesis of POP1-PO$_3$H$_2$ (9): The synthetic procedures are similar to that of POP2-PO_3H_2 with the yield of each individual step higher than 95%.

Synthesis of POP3-PO$_3$H$_2$

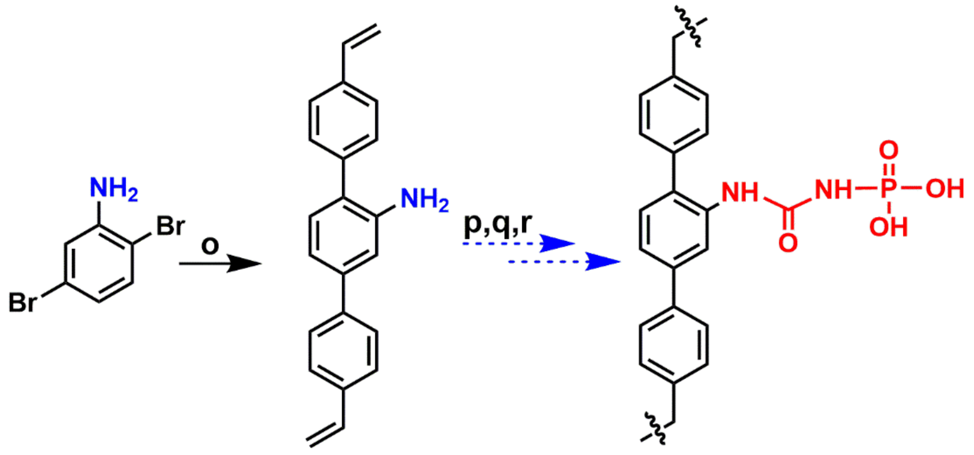

(10) (11)

Reagents: (o) (4-vinylphenyl)boronic acid, Pd(OAc)$_2$; (p,q,r) AIBN, DMF; OCN-P(O)(OEt)$_2$; Me_3SiBr

***Synthesis of 4,4''-divinyl-[1,1':4',1''-terphenyl]-2'-amine (10)**:* 2,5-dibromoaniline (2.5 g, 10 mmol), (4-vinylphenyl)boronic acid (3.6 g, 24 mmol), K_2CO_3 (5.52 g, 40 mmol), and Pd(PPh$_3$)$_4$ (275 mg, 0.25 mmol) were dissolved in a mixture of toluene (25 mL), THF (25 mL), and H_2O (5 mL), and the resulting mixture was refluxed at 90 °C under N_2 atmosphere for 36 h. The residue was extracted with ethyl acetate, washed with brine, dried over Na_2SO_4, and evaporated under reduced pressure, giving the crude compound which was purified by flash chromatography with hexane/ethyl acetate (5:1) as eluent to afford the title compound as a white solid. Yield: 2.64 g (89%). ^{1}H NMR (400 MHz, $CDCl_3$, 298K, TMS): δ 7.58 (d, 2H, J=6 Hz), 7.47-7.52 (m, 6H), 7.21 (d, 1H, *J*=6.4 Hz), 7.07 (d, 1H, J=6.4 Hz), 7.00 (s, 1H), 6.73-6.80 (m, 2H), 5.78-5.83 (m, 2H), 5.28 (t, 2H, *J*=8.4), 3.85 (s, 2H) ppm. ^{13}C NMR (125 MHz, $CDCl_3$, 298K, TMS) 143.86, 141.14, 140.41, 138.67, 136.64, 136.56, 136.47, 136.42, 130.85, 129.23,127.13, 126.72, 126.61, 126.44, 117.56, 114.11, 114.08, 113.86 ppm.

Synthesis of POP3-PO$_3$H$_2$ (11): The synthetic procedures are similar to that of POP2-PO$_3$H$_2$ and POP1-PO$_3$H$_2$ with the yield of each individual step higher than 95%.

Sorption Experiments

The aqueous solutions with different uranium concentrations were obtained by diluting the stock $UO_2(NO_3)_2 \cdot 6H_2O$ solution with the proper amount of distilled water unless otherwise indicated. The pH values of the solutions were adjusted by HNO_3 or NaOH aqueous solution. The concentrations of uranium during all the experiments were detected by inductively coupled plasma-optical emission spectroscopy (ICP-OES) and inductively coupled plasma-mass spectrometry (ICP-MS) for extra-low uranium concentrations. All the adsorption experiments were performed at ambient conditions.

K_d value calculation. The distribution coefficient (K_d) value as used for the determination of the affinity and selectivity of sorbents for UO_2^{2+}, is given by the equation:

$$K_d = (\frac{C_0 - C_e}{C_e}) \times \frac{V}{m}$$

where V is the volume of the treated solution (mL), m is the amount of adsorbent (g), C_0 is the initial concentration of uranium, and C_e is the equilibrium concentration of uranium. In the present work, the K_d values were measured in the presence of two equivalents of immobilized ligands in various adsorbents (2.3 mg, 2.2 mg, and 3.6 mg for POP1-PO_3H, POP2-PO_3H, and POP3-PO_3H, respectively) used against one equivalent of uranyl in the corresponding amount of aqueous solutions (10 ppm, 100 mL). To guarantee the adsorptions reached equilibrium, an overnight stirring step was used.

Uranium sorption isotherms. To obtain the uranium adsorption isotherms for various adsorbents, 5 mg of each was added into 10 mL aqueous solutions with different concentrations of uranium. Sorbent materials were suspended fully by brief sonication, and then the mixtures were stirred vigorously overnight, by which time it was assumed that adsorption equilibrium had been reached. The treated solutions were filtrated through a 0.45-μm membrane filter. The supernatant was analyzed using ICP analysis to determine the remaining uranium concentration. The adsorbed amount at equilibrium (q_e, mg g^{-1}) was calculated by:

$$q_e = \frac{(C_0 - C_e) \times V}{m}$$

where V is the volume of the treated solution (mL), m is the amount of used adsorbent (g), and C_0 and C_e are the initial concentration and the final equilibrium concentration of uranium, respectively.

Uranium sorption kinetics from distilled water. Uranium aqueous solution (400 mL, 20 ppm) and adsorbents (5 mg) were added to an Erlenmeyer flask with a magnetic stir bar. The mixture was stirred at room temperature. At appropriate time intervals, aliquots (5 mL) were taken from the mixture, and the adsorbents were separated by a syringe filter (0.45-μm membrane filter). The uranium concentrations in the resulting solutions were analyzed by ICP-OES. The adsorption capacity at different intervals was calculated as follows:

$$\text{Adsorption capacity (mg/g)} = (C_0 - C_t) \times V/m$$

where V is the volume of the treated solution (mL) and m is the amount of used adsorbent (mg), and C_i and C_t are the initial concentration and the concentration of uranium at t (min), respectively.

Uranium sorption kinetics from potable water. Uranium aqueous solution (200 mL, 5 ppm), and adsorbents (5 mg) were added to an Erlenmeyer flask with a magnetic stir bar. The mixture was stirred at room temperature for 3 h. At appropriate time intervals, the aliquots (3 mL) were taken from the mixture, and the adsorbents were separated by a syringe filter (0.45 μm membrane filter). The uranium concentrations in the resulting solutions were analyzed by ICP-MS. The percentage removal of uranium was calculated as follows:

$$\text{Removal percentage (\%)} = \frac{C_0 - C_t}{C_0} \times 100$$

Selectivity tests. To evaluate the removal efficiency of these adsorbents towards uranium species in the presence of a large excess of competing ions, tests were performed using a distilled water sample (100 mL) containing uranium (*ca.* 5 ppm) and various ions (Cu^{2+}, Fe^{3+}, Co^{2+}, Pb^{2+}, Zn^{2+}, La^{3+}, Ce^{3+}, Cs^{+}, Sr^{2+}, Mg^{2+}, and Ca^{2+}) with nearly equal concentrations (*ca.* 100 ppm) at a phase ratio (V/m) of 100 mL g^{-1}. After being stirred at room temperature for a certain time, aliquots were taken from the mixture, and the adsorbents were separated by a syringe filter (0.45 μm membrane filter). The uranium concentrations in the resulting solutions were analyzed by ICP-MS.

Uranium sorption kinetics from simulated seawater. Simulated seawater (25.6 g L^{-1} NaCl and 0.198 g L^{-1} $NaHCO_3$) spiked with 15 ppm uranium (400 mL), and adsorbents (5 mg) were added to an Erlenmeyer flask with a magnetic stir bar. The mixture was stirred at room temperature, and at appropriate time intervals, aliquots (5 mL) were taken from the mixture, and the adsorbents were separated by syringe filter (0.45 μm membrane filter). The uranium concentrations in the resulting solutions were analyzed by ICP-OES.

Uranium removal kinetics from simulated seawater. Simulated seawater (25.6 g L^{-1} NaCl and 0.198 g L^{-1} $NaHCO_3$) spiked with 4056 ppb uranium (10 mL), and adsorbents (5 mg) were added to each 20 mL vial with a magnetic stir bar. The mixtures were stirred at room temperature. At appropriate time intervals, the adsorbents were separated by a syringe filter (0.45 μm membrane filter). The uranium concentrations in the resulting solutions were analyzed by ICP-MS.

Uranium enrichment from real seawater. Adsorbent material (5 mg) was immersed in a tank containing 5 gallons of seawater and shaken at 100 rpm at room temperature. After 56 days, the adsorbent was collected by filtration, washed with water, and dried at 80 °C under vacuum for 24 h. The amount of uranium enriched in the adsorbent was determined by ICP-MS analysis after being digested by aqua regia.

Methods

Materials and measurements. Commercially available reagents were purchased in high purity and used without purification. ^{1}H NMR spectra were recorded on a Bruker Avance-400 (400 MHz) spectrometer. Chemical shifts are expressed in ppm downfield from TMS at δ=0 ppm, and J values are given in Hz. ^{13}C (100.5 MHz) cross-polarization magic-angle spinning (CP-MAS) NMR experiments were recorded on a Varian infinity plus 400 spectrometer equipped with a magic-angle spin probe in a 4-mm ZrO_2 rotor. Nitrogen sorption isotherms at the temperature of liquid nitrogen were measured using Micromeritics ASAP 2020M and Tristar system. The samples were outgassed for 1000 min at 80 °C before the measurements. Scanning electron microscopy (SEM) and energy dispersive X-ray spectroscopy (EDX) mapping were performed on a Hitachi SU 8000. Transmission electron microscope (TEM) images were collected using a Hitachi HT-7700 or JEM-2100F field emission electron microscope (JEOL, Japan) with an acceleration voltage of 110 kV. XPS spectra were performed on a Thermo ESCALAB 250 with Al Kα irradiation at θ=90° for X-ray sources, and the binding energies were calibrated using the C1s peak at 284.9 eV. IR spectra were recorded on a Nicolet Impact 410 FTIR spectrometer. ICP-OES was performed on a Perkin-Elmer Elan DRC II Quadrupole. ICP-MS was performed on a Perkin-Elmer Elan DRC II Quadrupole Inductively Coupled Plasma Mass Spectrometer.

Theoretical methods:

Quantum chemical calculations. Density functional theory (DFT) calculations were performed with the Gaussian 16, Revision B.01 program package[1] using the M06[2] functional with the standard Stuttgart small-core (SSC) 1997 relativistic effective core potential (RECP)[3] and the associated contracted [8s/7p/6d/4f] basis set for uranium atom, along with the 6-311++G(d,p) basis set for the light atoms. Frequency calculations were performed at the B3LYP/SSC/6-31+G(d)[4] level to confirm that geometries (optimized at the same B3LYP/SSC/6-31+G(d) level) were minima on the potential energy surface. All thermal corrections to the Gibbs free energy were computed using the ideal gas molecular partition functions within the rigid-rotor quasiharmonic oscillator approximation. In this approximation,[5] vibrational frequencies lower than 30 cm^{-1} were raised to 30 cm^{-1} due to the breakdown of the harmonic oscillator approximation for low frequency modes. Using the gas-phase geometries, implicit solvent corrections were obtained at 298 K with the SMD[6] model as implemented in Gaussian 09 at the B3LYP/SSC/6-31+G(d) level of theory. The results are reported using the lowest energy complexes identified at the M06/SSC/6-311++G(d,p) level for a given stoichiometry and binding motif. The preference for using a combination of the M06 and the B3LYP functionals with the SMD solvation model was based on the results of our previous studies.[7,8]

Ligand-UO_2^{2+} interactions. Assessment of second-order stabilization energies ($E^{(2)}$, kcal/mol) in the considered uranyl complexes was performed with the natural bond orbital (NBO) method[9] at M06/SSC/6-311++G(d,p) using commercial stand-alone NBO 6.0 program.[10] The donor-acceptor interaction energy (second-order stabilization energies ($E^{(2)}$) in the NBOs was estimated via second-order perturbation theory analysis of the Fock matrix.[9] For each donor orbital (i) and acceptor orbital (j), the stabilization energy $E^{(2)}$ associated with $i \rightarrow j$ delocalization is given by:

$$E_{i,j}^{(2)} = -o_i \frac{\langle i | \hat{F}_{(i,j)} | j \rangle^2}{\varepsilon_j - \varepsilon_i}$$

, where o_i is the donor orbital occupancy, $\hat{F}_{(i,j)}$ is the Fock operator, and ε_i and ε_j are the orbital energies.

Thermodynamic analysis of complexation. Complexation free energies in aqueous solution, ΔG_{aq}, and stability constants, log β, were calculated using the methodology described in our previous works on uranium complexes. More specifically, this approach utilizes a thermodynamic cycle scheme involving the calculation of the gas-phase free energies and the change in free energy upon transfer of 1 mole of a species from the gas to the aqueous phase under standardized conditions. Once the various free energy terms of the cycle are calculated using quantum chemical methods, then the change in free energy for the aqueous reaction (ΔG_{aq}) can be determined. The log β values are obtained from the relation of log β to ΔG_{aq} by the following equation:

$$\log \beta = -\frac{\Delta G_{aq}}{2.303\,RT}$$

The final log β values in the main text are reported after applying the corresponding regression equation (log β^{expt} = 0.5693×log β^{calc})[8] to the calculated log β. Furthermore, a correction factor of 2.2 log units has been applied to correct log β of uranyl complex with the more flexible ligand in complex **2** (main text, Figure 4b), because the calculations seem to underestimate entropy of freely rotatable bonds using a harmonic oscillator approximation. This was based on the predictions of log β values for the uranyl complexes with simple dicarboxylic acids having different lengths of alkyl chain connecting two carboxylate groups. As evident from Tables S5 and S6, our approach significantly overestimates the formation constant for the more flexible ligands (adipic and pimelic acids).

EXAFS:

The X-ray absorption data were collected at Beamline 1W1B at the Beijing Synchrotron Radiation Facility (BSRF), Institute of High Energy Physics (IHEP), Chinese Academy of Sciences (CAS). Spectra were collected at the uranium L_3-edge (17166 eV) in transmission mode. The X-ray white beam was monochromatized by a double crystal Si(111) monochromator and detuned by 20% to reduce the contribution of higher-order harmonics to below the level of noise. The K-edge of Yttrium foil (17038 eV) was used as the reference for energy calibration and measured simultaneously for all samples. All spectra were collected at room temperature.

Samples were centered on the beam and adjusted to find the most homogeneous location in the sample for data collection. Data were collected over eight regions: -200 to -30 eV (4 eV step size, dwell time of 0.5 seconds), -30 to -17 eV (2 eV step size, dwell time of 0.5 seconds), -17 to 50 eV (1.2 eV step size, dwell time of 0.7 seconds), 50 to 150 eV (2 eV step size, dwell time of 0.9 seconds), 150 to 300 eV (2 eV step size, dwell time of 1 seconds), 300 to 600 eV (3 eV step size, dwell time of 2.0 seconds), 600 to 800 eV (4 eV step size, dwell time of 2.0 seconds), 800 to 840 eV (5 eV step size, dwell time of 3.0 seconds),Three scans were collected for each sample.

The data were processed and analyzed using the Athena and Artemis programs of the IFEFFIT package. Reference foil data were aligned to the first zero-crossing of the second derivative of the normalized μ(E) data, which was subsequently calibrated to the literature E0 for the yttrium K-edge (17038 eV). Spectra were averaged in μ(E) prior to normalization.

Table S1. The P species content in various samples and corresponding uranium uptake capacities.

Sample	Theoretical values (wt.%)	Elemental analysis (wt.%)	Experimental uranium uptake capacity (mg g^{-1})[a]	Coordination number (Ligand/U)[b]
POP1-PO_3H_2	11.9	11.7	571	1.55
POP2-PO_3H_2	12.7	12.3	502	1.86
POP3-PO_3H_2	7.3	6.9	398	1.31

[a]The uptake capacity of uranium was evaluated under the conditions with an initial uranium concentration of 20 ppm at a phase ratio (V/m) of 80000 mL g^{-1}. [b]The coordination number of the ligands in the adsorbents to each uranyl ions was calculated based on the number of ligands in the adsorbent to the number of the uranium species captured.

Table S2. Concentrations of metal ions before and after the treatment of POP2-PO_3H_2.

Solution	Concentration (ppm)									
	U	Ce	La	Zn	Co	Fe	Cu	Pb	Na	Sr	Ca
Before treatment	5	100	100	100	100	100	100	100	100	100	100
After treatment	0.0087	20.3	10.2	99.7	95.2	96.7	95.7	99.9	>99.9	>99.9	99.9
recycle 1	0.0086	20.5	10.1	99.8	93.4	96.2	95.9	99.8	>99.9	>99.9	99.9
recycle 2	0.0088	21.4	10.3	99.8	95.1	96.9	95.8	99.9	>99.9	>99.9	99.9
recycle 3	0.0091	20.8	9.8	99.7	94.7	96.4	96.1	99.9	>99.9	>99.9	99.9
recycle 4	0.0088	20.6	10.1	99.8	94.9	96.5	96.2	99.8	>99.9	>99.9	99.9

Conditions: Tests were performed using a distilled water sample (50 mL) containing uranium (5 ppm) and various ions (Cu^{2+}, Fe^{3+}, Co^{2+}, Pb^{2+}, Zn^{2+}, La^{3+}, Ce^{3+}, Cs^{+}, Sr^{2+}, Mg^{2+}, and Ca^{2+}) with equal concentrations (100 ppm) at a phase ratio (V/m) of 100 mL g^{-1}. The adsorbent was regenerated by using HNO_3 (1 M) as eluent.

Table S3. Uranium sorption performance of representative adsorbents in the literature.

Adsorbents	Water (mg g^{-1})	Artificial seawater (mg g^{-1})	Seawater (mg g^{-1})
poly(imide dioxime) nanofiber[a]	951	--	8.7
**This work (POP2-PO_3H_2)**	**501**	**304**	**5.01**
POP-*o*NH_2-AO[b]	530	290	4.36
p(2DVB-VBC)-2PAN[c]	--	80	1.99
S_x-LDH[d]	330	--	0.00072
$K_2MnSn_2S_6$ (KMS-1)[e]	382	--	0.00029
zero-valent iron[f]	2400	(50-300 ppm) 240-1410	--
MIL-101-DETA[g]	350		--
FJSM-SnS[h]	338	--	--
S_x-LDH[i]	330		--
(MIL-101(Cr)-triazole-COOH[j]	304		--
MOF-76[k]	298		--
ND-AO[l]	212	121	--
MSPh-III (phosphonic acid-modified mesoporous material)[m]	182	66.7	--
V_2CT_x[n]	174	377	--
Mesoporous Carbon Materials[o]	97	67	--
KIT-6-80-P[p]	56		--
Am-p(AN-c-MAc) particles[q]	51.5	--	--
Am-p(AN-c-MAc)[r]	51.5		--
PAO/PVDF[s]	--	1.6	--
S-CP40[*E]-AO[t]	--	57	--
AF series adsorbents[o]		200	--
F-AA2 fiber[p]		50	--

[a]D. Wang, J. Song, J. Wen, Y. Yuan, Z. Liu, S. Lin, H, Wang, H. Wang, S. Zhao, X. Zhao, M. Fang, M. Lei, B. Li, N. Wang, X. Wang, H. Wu, *Adv. Energy Mater.* **2018**, *26*, 1802607.

[b]Q. Sun, B. Aguila, J. Perman, A. S. Ivanov, V. S. Bryantsev, L. D. Earl, C. W. Abney, L. Wojtas, S. Ma, *Nat. Commun.* **2018**, *9*, 1644.

[c]Y. Yue, R. T. Mayes, J. Kim, P. F. Fulvio, X.-G. Sun, C. Tsouris, J. Chen, S. Brown, S. Dai, *Angew. Chem. Int. Ed.* **2013**, *52*, 13458.

[d]S. Ma, L. Huang, L. Ma, Y. Shim, S. M. Islam, P. Wang, L.-D. Zhao, S. Wang, G. Sun, X. Yang, M. G. Kanatzidis, *J. Am. Chem. Soc.* **2015**, *137*, 3670.

[e]M. J. Manos, M. G. Kanatzidis, *J. Am. Chem. Soc.* **2012**, *134*, 16441.

[f]L. Ling, W.-x. Zhang, *J. Am. Chem. Soc.* **2015**, *137*, 2788.

[g]Z.-Q. Bai, L.-Y. Yuan, L. Zhu, Z.-R. Liu, S.-Q. Chu, L.-R. Zheng, J. Zhang, Z.-F. Chai, W.-Q. Shi, *J. Mater. Chem. A* **2015**, *3*, 525.

[h]M.-L. Feng, D. Sarma, X.-H. Qi, K.-Z. Du, X.-Y. Huang, M. G. Kanatzidis, *J. Am. Chem. Soc.* **2016**, *138*, 12578.

[i]S. Ma, L. Huang, L. Ma, Y. Shim, S. M. Islam, P. Wang, L.-D. Zhao, S. Wang, G. Sun, X. Yang, M. G. Kanztzidis, *J. Am. Chem. Soc.* **2015**, *13*, 3670.

[j]L. Li, W. Ma, S. Shen, H.-F. Shi, L.-Y. Yuan, T. Tian, Z.-F. Chai, H. Wang, Z.-M. Sun, *Chem. Commun.* **2013**, *49*, 10415.

[k]Y. Li, L. Wang, B. Li, M. Zhang, R. Wen, X. Guo, X. Li, J. Zhang, S. Li, *ACS Appl. Mater. Interfaces* **2016**, *8*, 28853.

[l]J. L. Vivero-Escoto, M. Carboni, C. W. Abney, K. E. deKrafft, W. Lin, *Micropor. Mesopor. Mater.* **2013**, *180*, 22.

[m]L. Wang, L. Yuan, K. Chen, Y. Zhang, Q. Deng, S. Du, Q. Huang, L. Zheng, J. Zhang, Z. Chai, M. W. Barsoum, X. Wang, W. Shi, *ACS Appl. Mater. Interfaces* **2016**, *8*, 16396.

[n]M. Carboni, C. W. Abney, K. M. L. Taylor-Pashow, J. L. Vivero-Escoto, W. Lin, *Ind. Eng. Chem. Res.* **2013**, *52*, 15187.

[o]P. J. Lebed, J.-D. Savoie, J. Florek, F. Bilodeau, D. Larivière, F. Kleitz, *Chem. Mater.* **2012**, *24*, 4166.

[p]N. Sahiner, H. Yu, G. Tan, J. He, V. T. John, D. A. Blake, *ACS Appl. Mater. Interfaces* **2012**, *4*, 163.

[q]S. Xie, X. Liu, B. Zhang, H. Ma, C. Ling, M. Yu, L. Li, J. Li, *J. Mater. Chem. A* **2015**, *3*, 2552.

[r]C. Gunathilake, J. Górka, S. Dai, M. Jaroniec, *J. Mater. Chem. A* **2015**, *3*, 11650.

[s]S. Das, Y. Oyola, R. T. Mayes, C. J. Janke, L.-J. Kuo, G. Gill. J. R. Wood, S. Dai, *Int. Eng. Chem. Res.* **2015**, *55*, 4110.

[t]S. Chatterjee, V. S. Bryantsev, S. Brown, J. C. Johnson, C. D. Grant, R. T. Matyes, B. P. Hay, S. Dai, T. Saito, *Int. Eng. Chem. Res.* **2015**, *55*, 4161.

Table S4. U—O and O--HOH distances (Å) in uranyl complexes shown in complex **1** and complex **2** (Figure S28) computed at the M06/SC/6-311++G(d,p) level of theory.

	complex **1**	complex **2**
U—O_1	2.284	2.287
U—O_3	2.307	2.315
U—O_{1w}	2.517	2.483
U—O_{2w}	2.546	2.552
U—O_{3w}	2.561	2.566
O_2--HO_{2w}H	1.780	1.776
O_4--HO_{3w}H	1.740	1.746
O_{1u}-HO_{1w}H	1.958	1.803
O_{2u}--HO_{1w}H	2.000	1.878

Table S5. Comparison of the strengths of ligand-uranyl interactions in complexes using natural bond orbital (NBO) method. Second-order stabilization energies ($E^{(2)}$, kcal mol^{-1}) indicate comparable uranyl binding affinities of **1** and **2** ligands.

Complex	Donor NBO → Acceptor NBO in UO_2^{2+} complexes (kcal mol^{-1})*			
	**LP_{O1}→n^*_U and LP_{O2}→n^*_U (phosphoryl oxygens)**	**LP_{O1w}→n^*_U (water oxygen)**	**LP_{O2w}→n^*_U (water oxygen)**	**LP_{O3w}→n^*_U (water oxygen)**
**1**	511	104	105	100
**2**	496	113	103	98

*The unstarred and starred labels correspond to Lewis (donor) and non-Lewis (acceptor) NBOs, respectively. Functional groups of the ligand contributing to the particular interaction are shown in parentheses. LP denotes an occupied lone pair; n^*_U denotes vacant U orbitals.

Table S6. Experimental and theoretically predicted log *β* values for uranyl complexes with dicarboxylate ligands depending on the length of the ligand's aliphatic chain.

ligand	log *β(expt)**	log *β (theor)*	absolute error
succinate	5.2	5.1	0.1
glutarate	4.8	4.8	0.0
adipicate	4.8	6.9	2.1
pimelicate	5.0	7.2	2.2

*Taken from *Martell, A. E.; Smith, R. M. Critical Stability Constant Database, 46* and corrected to zero ionic strength with the Davies equation.

Table S7. Simulating speciation diagrams.

$H^+ + OH^- \leftrightarrows H_2O$	14.00
$H^+ + NHCONHPO_3H^- \leftrightarrows NHCONHPO_3H_2$	11.6 (theor.)
$UO_2^{2+} + NHCONHPO_3H^- \leftrightarrows UO_2(NHCONHPO_3H_2)^+$	13.2 (theor.)
$UO_2^{2+} + 2\ NHCONHPO_3H^- \leftrightarrows UO_2(NHCONHPO_3H_2)_2$	22.8 (theor.)
$UO_2^{2+} + CO_3^{2-} + NHCONHPO_3H^- \leftrightarrows UO_2(CO_3)(NHCONHPO_3H^-)^-$	19.9 (theor.)
$UO_2^{2+} + CO_3^{2-} + 2NHCONHPO_3H^- \leftrightarrows UO_2(CO_3)(NHCONHPO_3H^-)_2^{2-}$	26.1 (theor.)
$UO_2^{2+} + OH^- \leftrightarrows UO_2(OH)^+$	5.25
$UO_2^{2+} + 2OH^- \leftrightarrows UO_2(OH)_2$	12.15
$UO_2^{2+} + 3OH^- \leftrightarrows UO_2(OH)_3^-$	20.25
$UO_2^{2+} + 4OH^- \leftrightarrows UO_2(OH)_4^{2-}$	32.40
$2UO_2^{2+} + OH^- \leftrightarrows (UO_2)_2(OH)^{3+}$	11.3
$2UO_2^{2+} + 2OH^- \leftrightarrows (UO_2)_2(OH)_2^{2+}$	22.4
$UO_2^{2+} + 2OH^- \leftrightarrows (UO_2)(OH)_2(s)$	-22.0
$UO_2^{2+} + CO_3^{2-} \leftrightarrows UO_2(CO_3)$	9.94
$UO_2^{2+} + 2CO_3^{2-} \leftrightarrows UO_2(CO_3)_2$	16.61
$UO_2^{2+} + 3CO_3^{2-} \leftrightarrows UO_2(CO_3)_3$	21.84

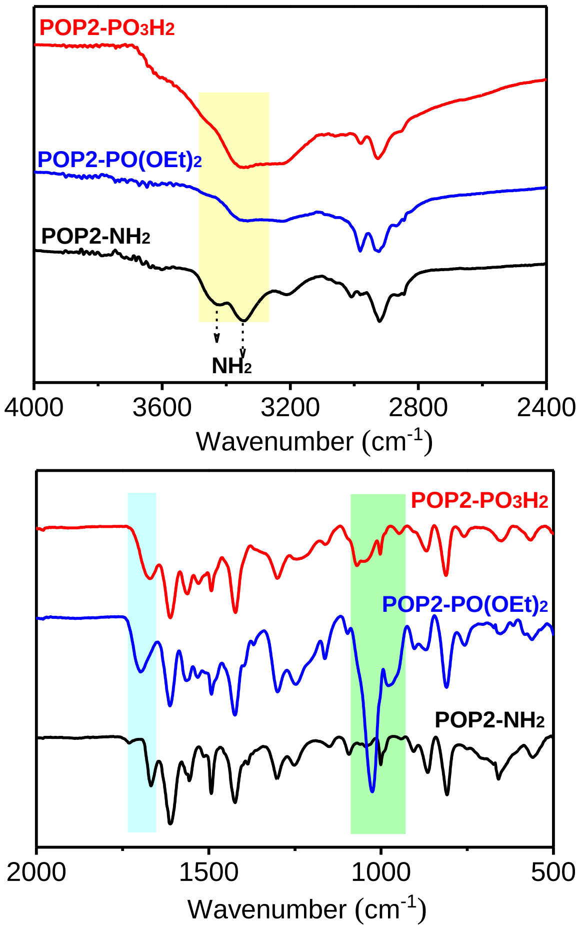

Figure S1. IR spectra of POP2-NH_2, POP2-$PO(OEt)_2$, and POP2-PO_3H_2.

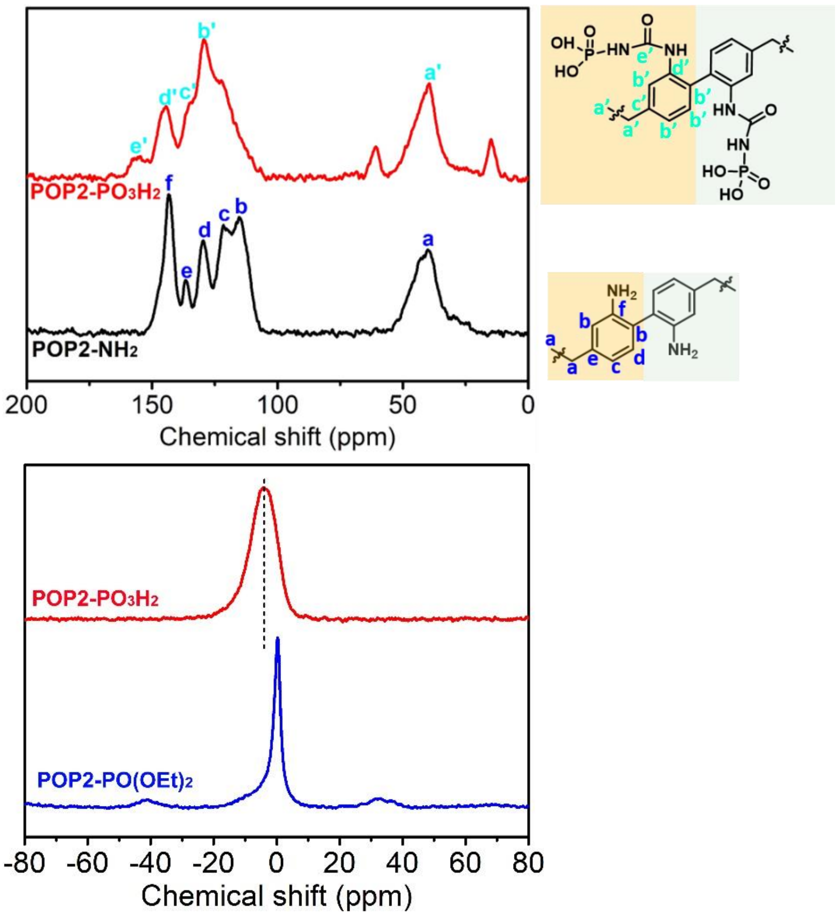

Figure S2. Solid-state NMR spectra of POP2-NH_2, POP2-$PO(OEt)_2$, and POP2-PO_3H_2. ^{13}C MAS NMR (top) and ^{31}P MAS NMR (bottom). The obvious chemical shift of P signals in POP2-$PO(OEt)_2$ and POP2-PO_3H_2 indicate the success of hydrolysis.

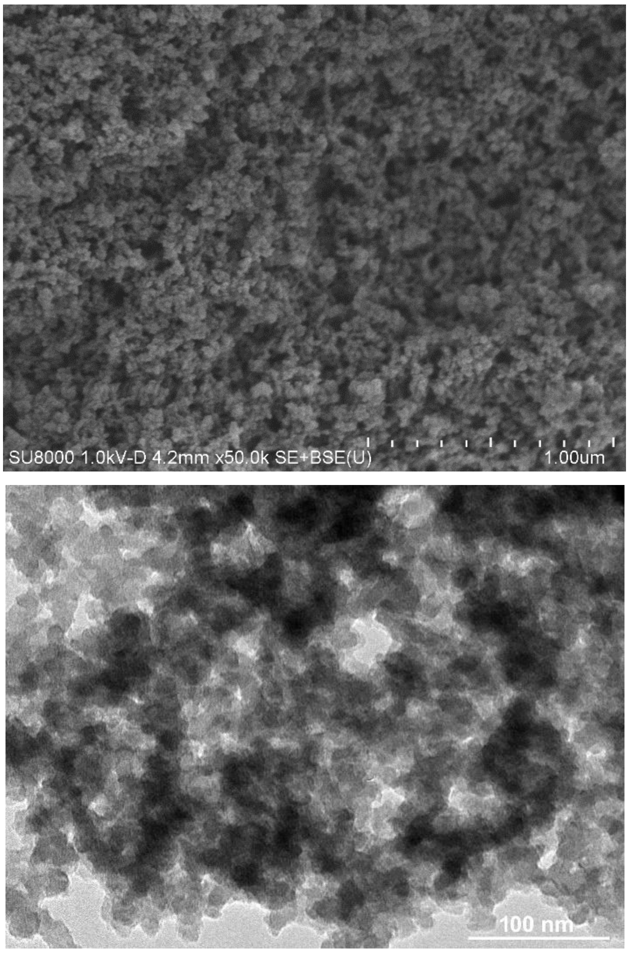

Figure S3. SEM (top) and TEM (bottom) images of POP2-NH_2.

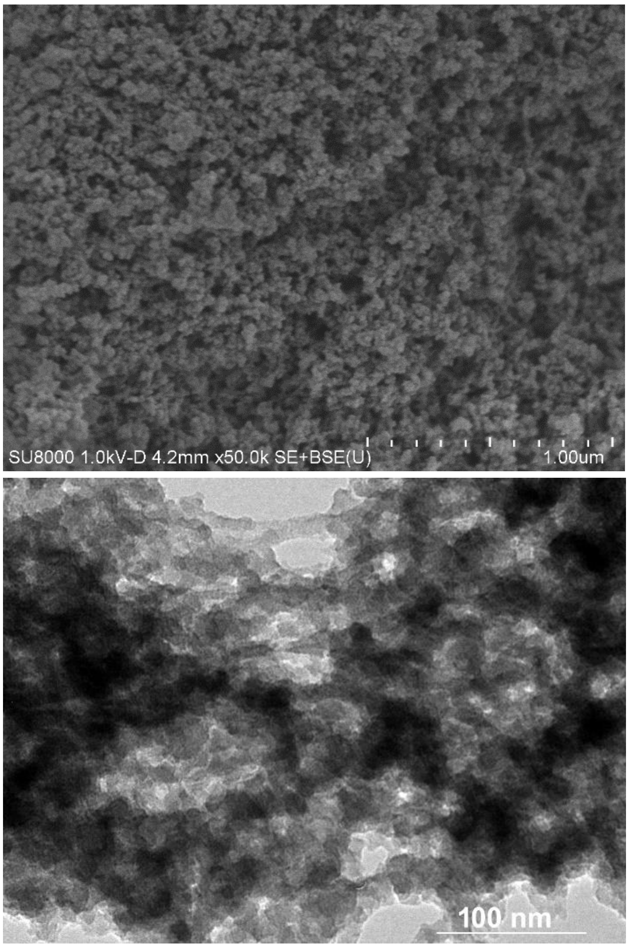

Figure S4. SEM (top) and TEM (bottom) images of POP2-PO_3H_2.

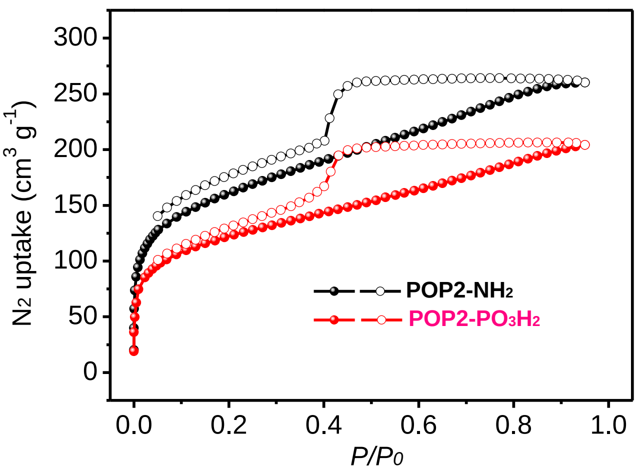

Figure S5. N_2 sorption isotherms for POP2-NH_2 and POP2-PO_3H_2 collected at 77 K with BET surface areas of 687 and 371 m^2 g^{-1}, respectively.

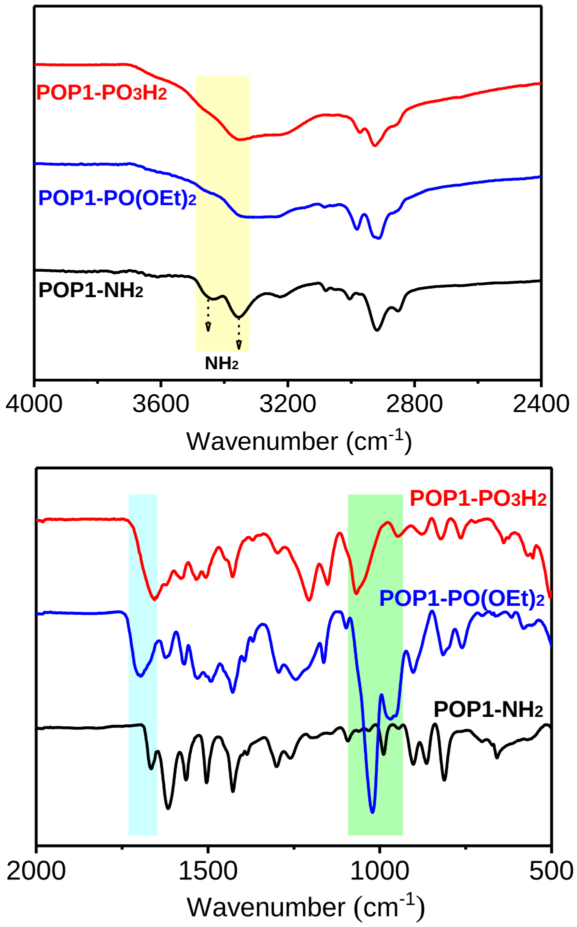

Figure S6. IR spectra of POP1-NH_2, POP1-$PO(OEt)_2$, and POP1-PO_3H_2.

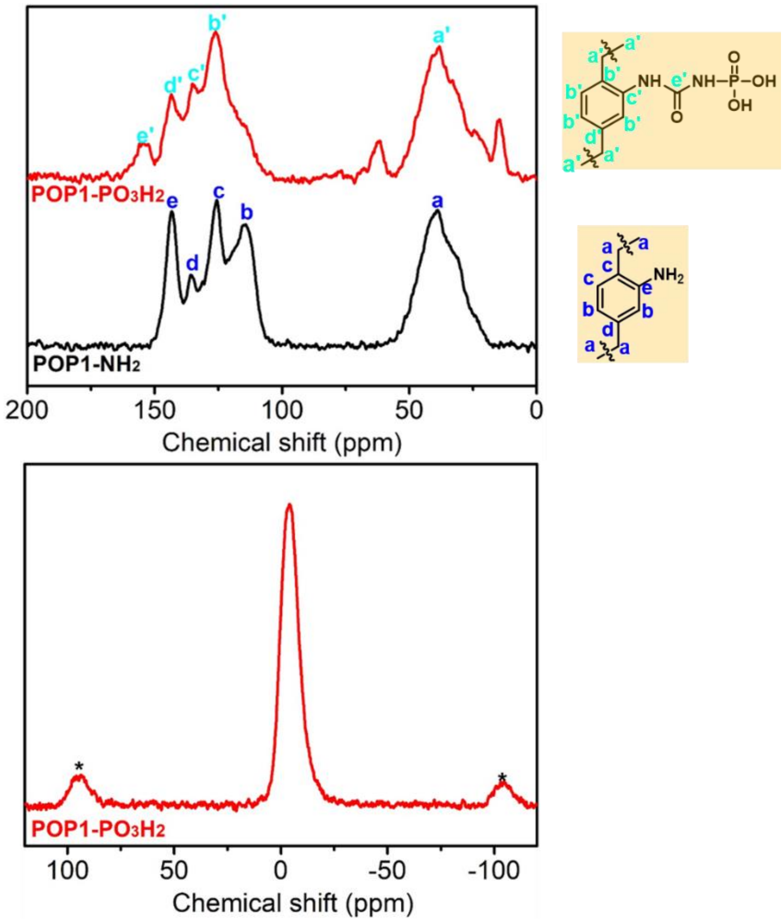

Figure S7. Solid-state NMR spectra of POP1-NH_2 and POP1-PO_3H_2. ^{13}C MAS NMR (top) and ^{31}P MAS NMR (bottom). *side band.

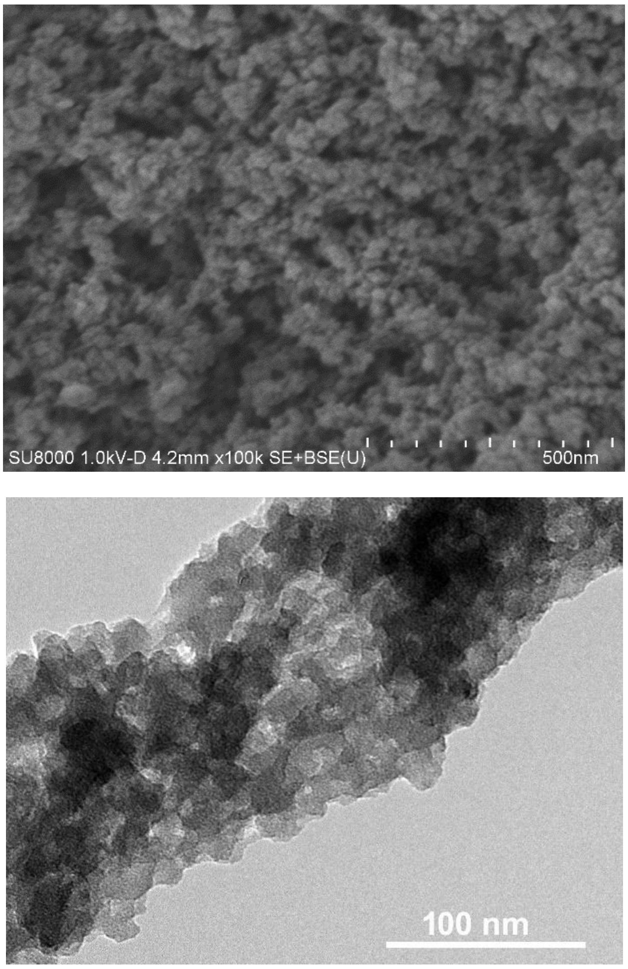

Figure S8. SEM (top) and TEM (bottom) images of POP1-NH_2.

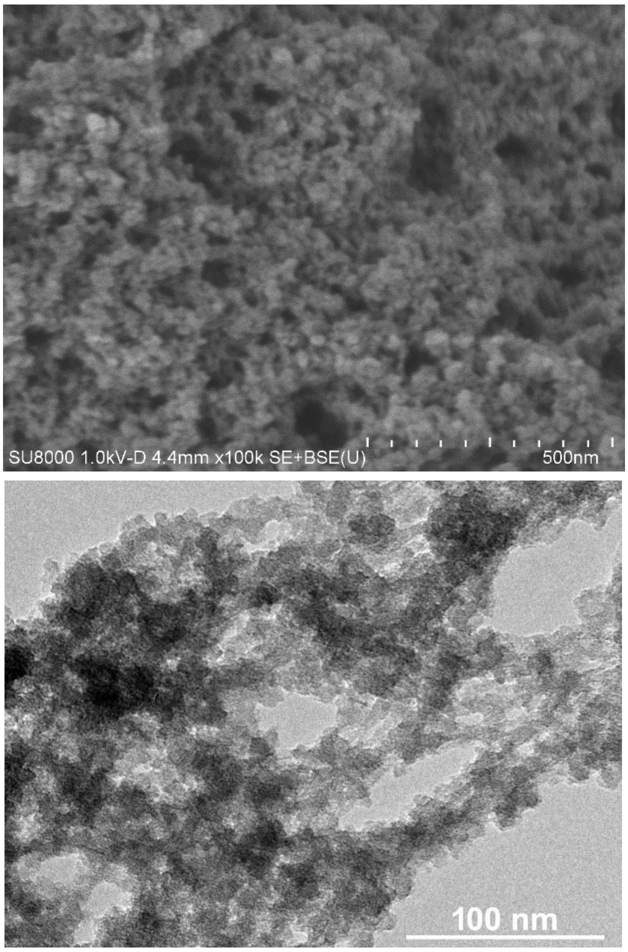

Figure S9. SEM (top) and TEM (bottom) images of POP1-PO_3H_2.

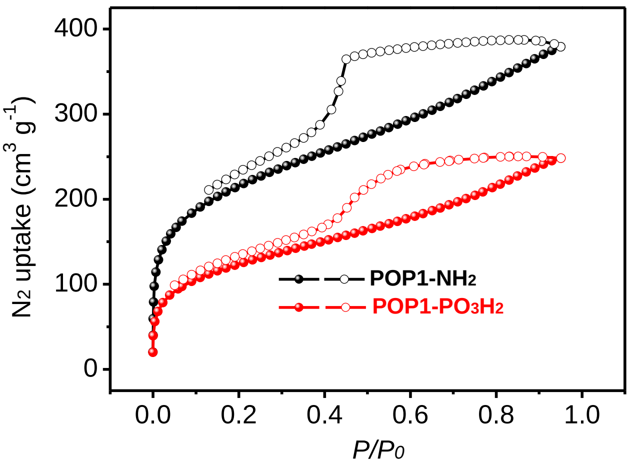

Figure S10. N_2 sorption isotherms for POP1-NH_2 and POP1-PO_3H_2 collected at 77 K with BET surface areas of 732 and 412 m^2 g^{-1}, respectively.

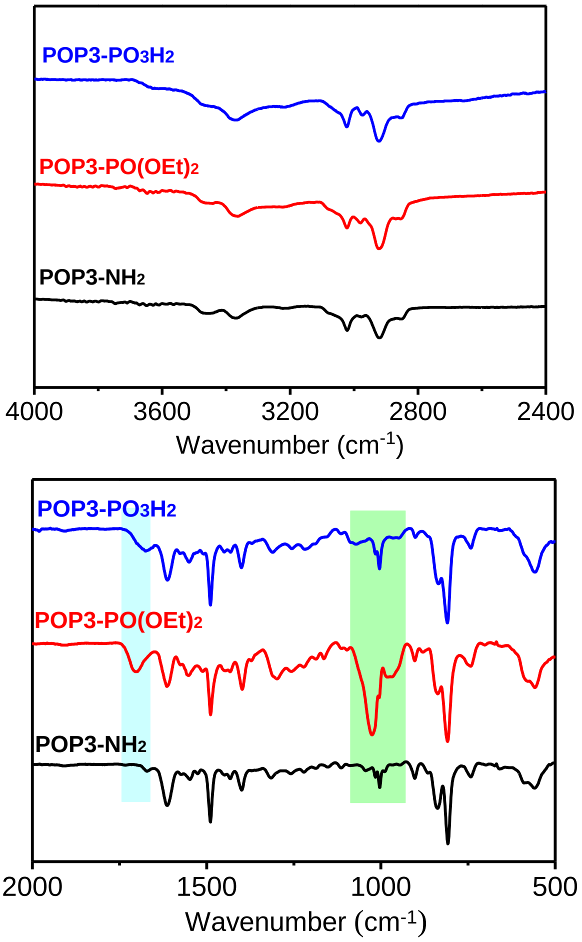

Figure S11. IR spectra of POP3-NH_2, POP3-$PO(OEt)_2$, and POP3-PO_3H_2.

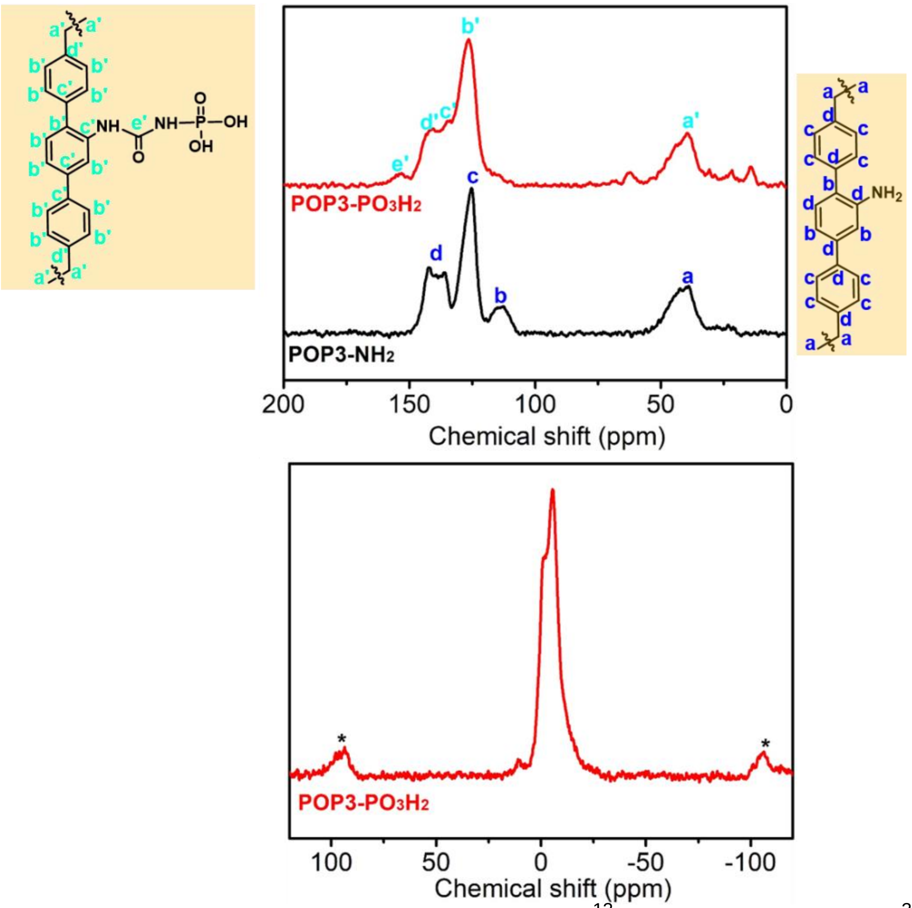

Figure S12. Solid-state NMR spectra of POP3-PO_3H_2. ^{13}C MAS NMR (top) and ^{31}P MAS NMR (bottom). *side band.

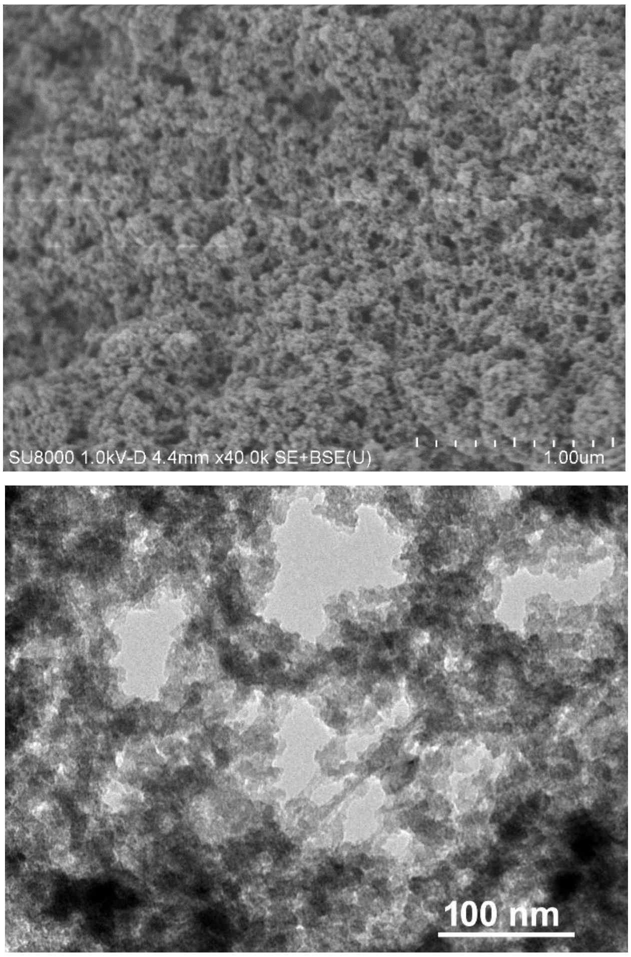

Figure S13. SEM (top) and TEM (bottom) images of POP3-NH_2.

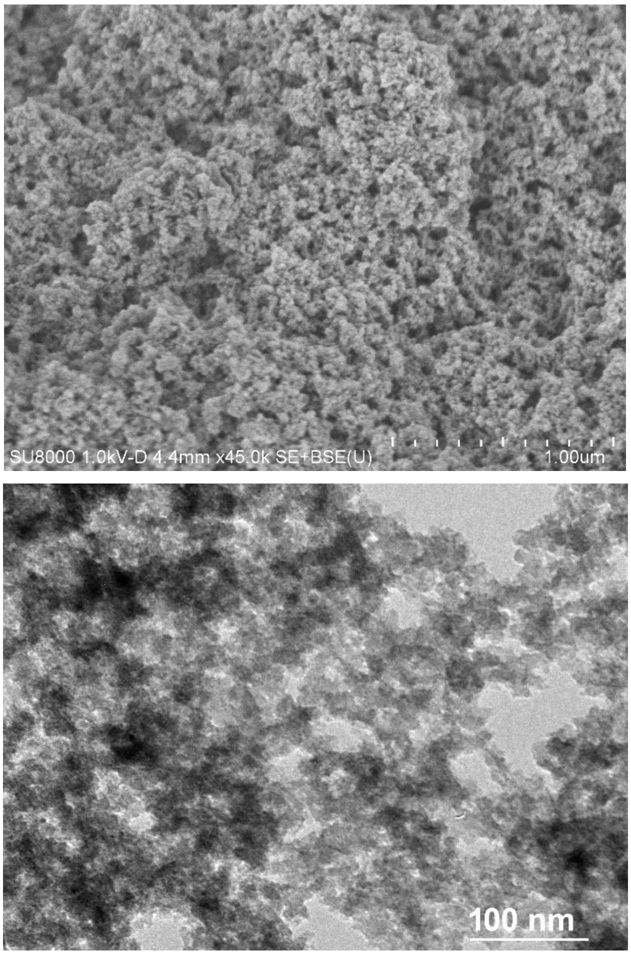

Figure S14. SEM (top) and TEM (bottom) images of POP3-PO_3H_2.

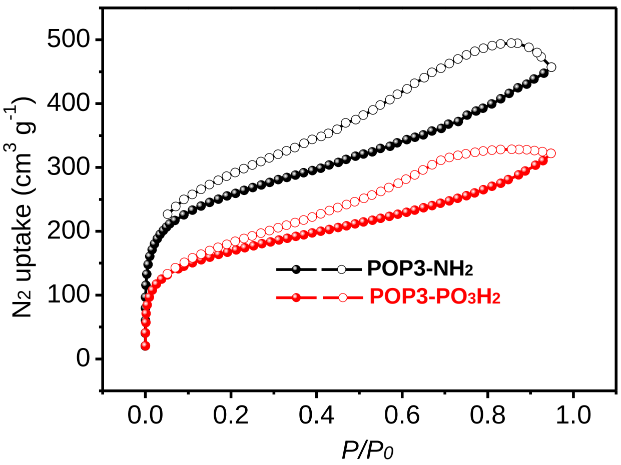

Figure S15. N_2 sorption isotherms for POP3-NH_2 and POP3-PO_3H_2 collected at 77 K with BET surface areas of 900 and 571 m^2 g^{-1}, respectively.

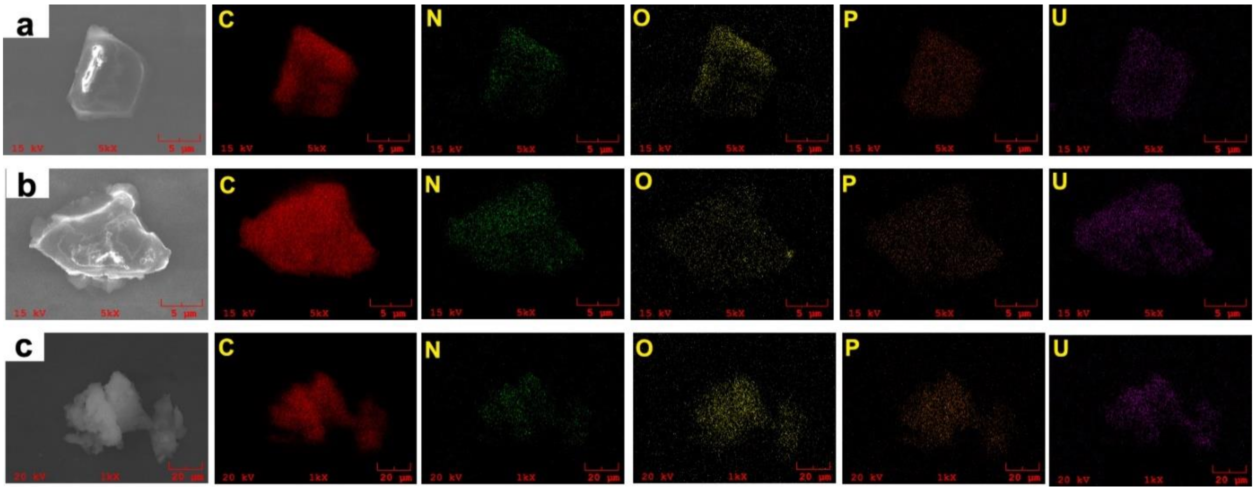

Figure S16. SEM images and corresponding EDX mapping. (a) U@POP1-PO_3H_2, (b) U@POP2-PO_3H_2, and (c) U@POP3-PO_3H_2.

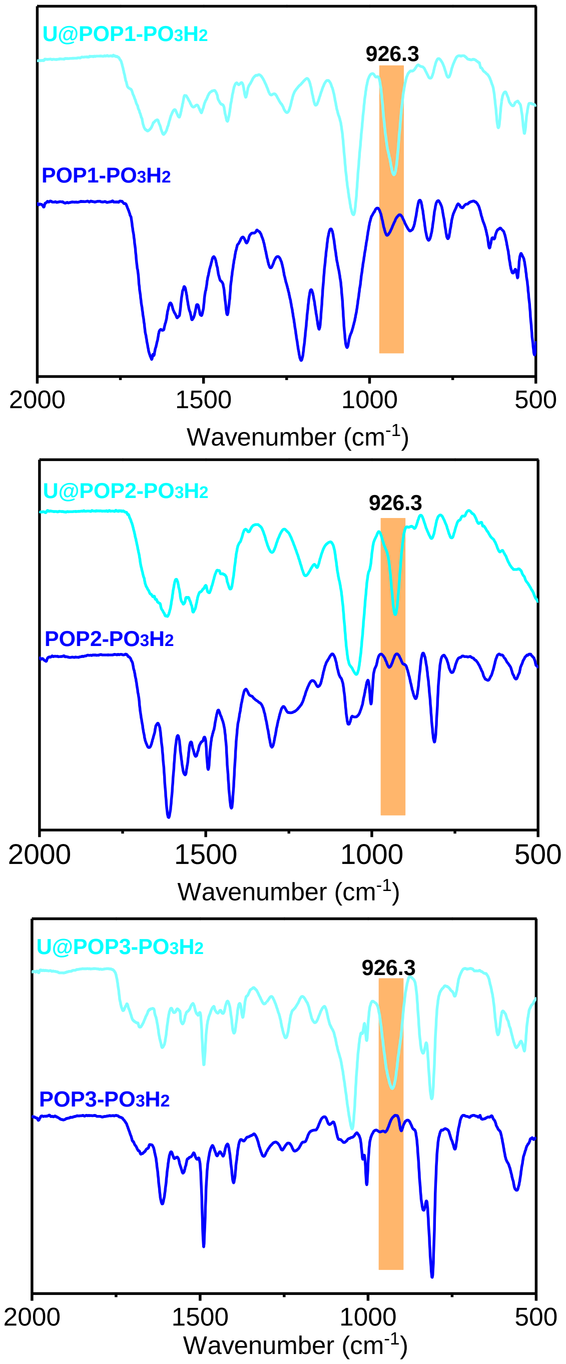

Figure S17. IR spectra. Peak that corresponds to the antisymmetric stretch vibrational mode of $[O=U=O]^{2+}$.

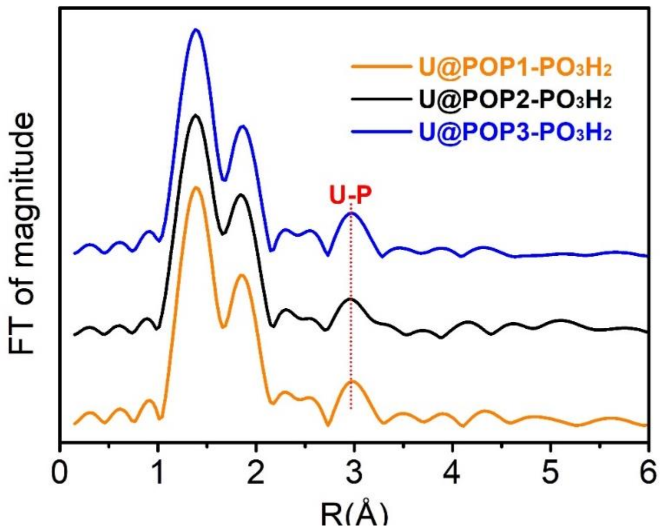

Figure S18. EXAFS spectra for U@POP1-PO_3H_2, U@POP2-PO_3H_2, and U@POP3-PO_3H_2.

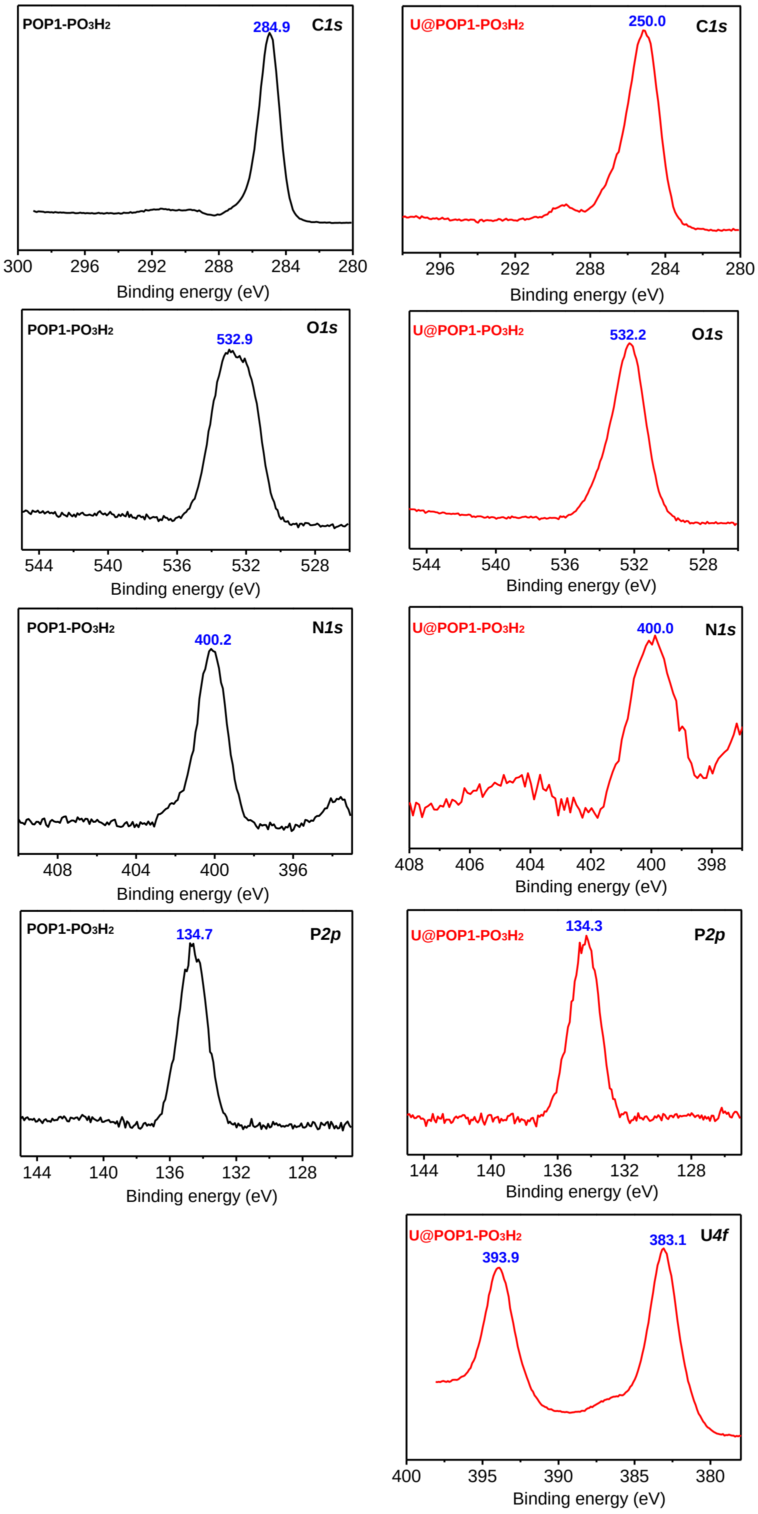

Figure S19. N*1s*, O*1s*, P*2p*, and U*4f* XPS spectra of POP1-PO_3H_2 and U@POP1-PO_3H_2.

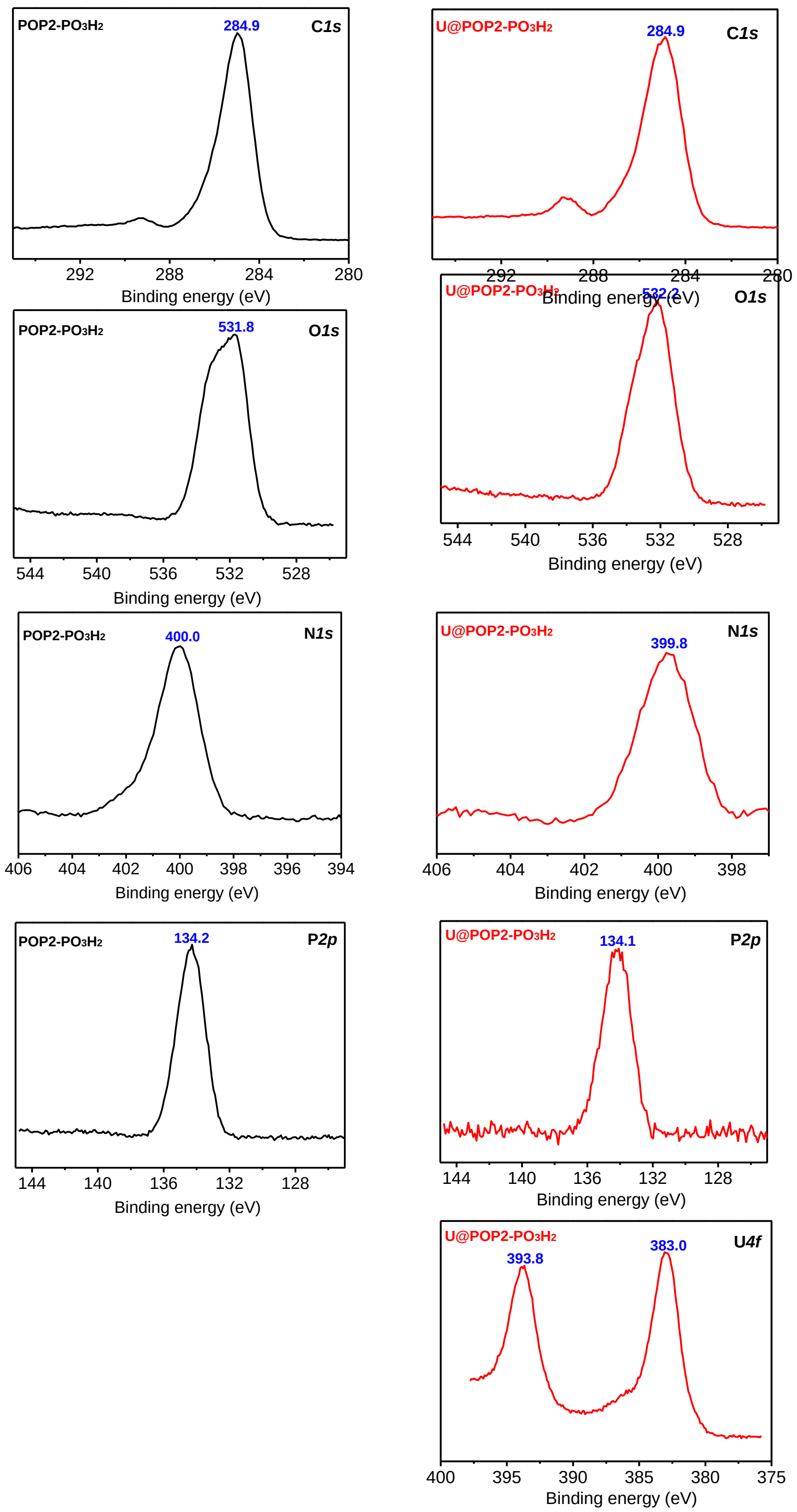

Figure S20. N*1s*, O*1s*, P*2p*, and U*4f* XPS spectra of POP2-PO_3H_2 and U@POP2-PO_3H_2.

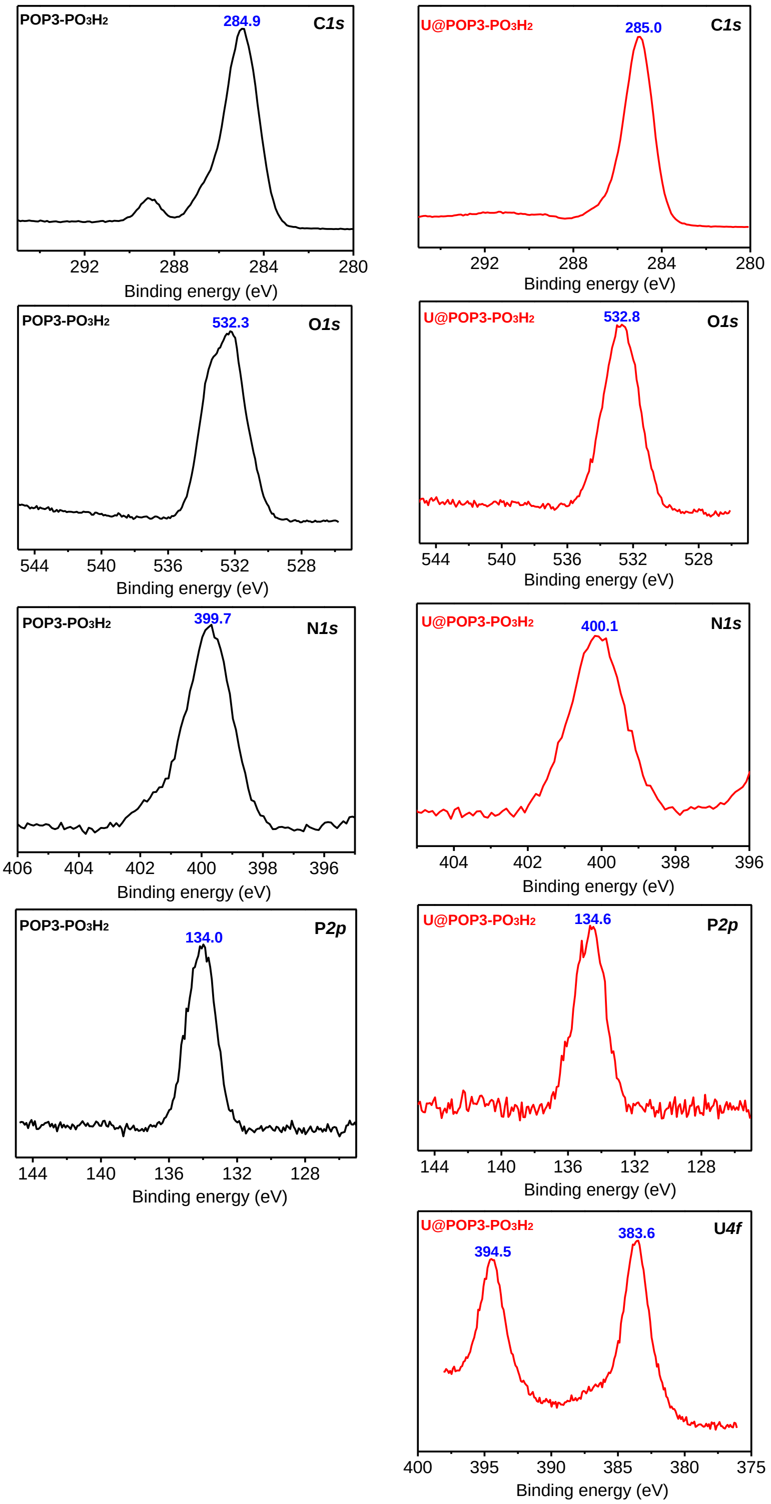

Figure S21. N*1s*, O*1s*, P*2p*, and U*4f* XPS spectra of POP3-PO_3H_2 and U@POP3-PO_3H_2.

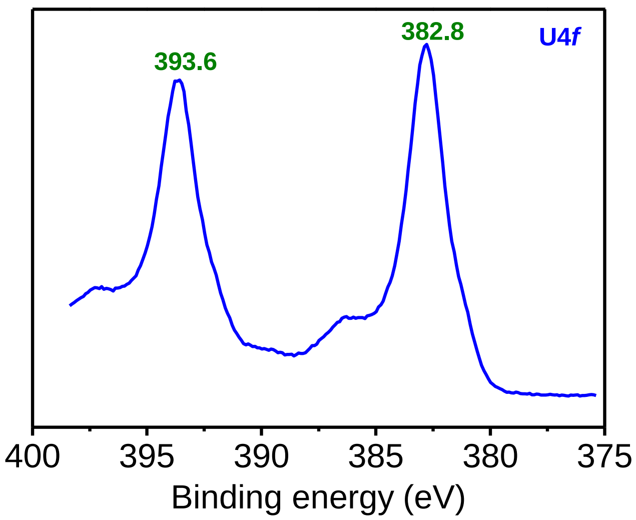

Figure S22. U4*f* XPS spectra of $UO_2(NO_3)_2{\cdot}6H_2O$.

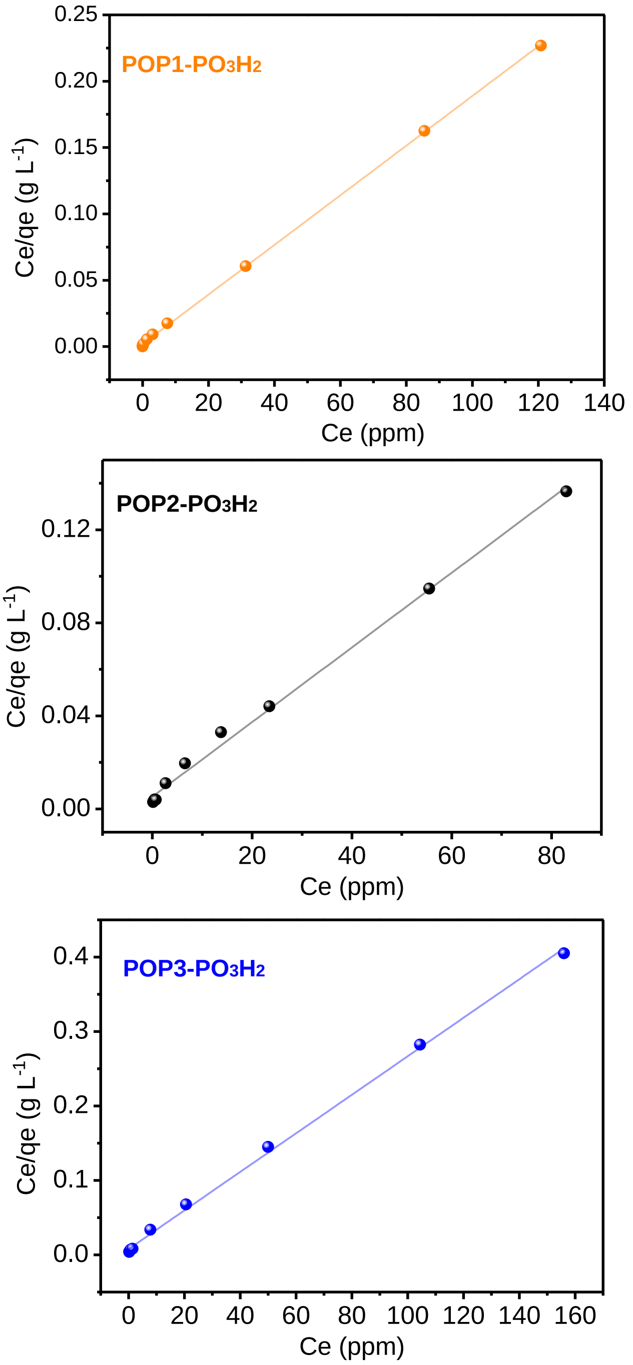

Figure S23. Linear regression by fitting the equilibrium data with the Langmuir adsorption model shown in Figure 3a of the main text. The equilibrium adsorption isotherm data were well fitted with Langmuir model expressed as: $q_e = (q_m K_a C_e)/(1 + K_a C_e)$, where C_e is the equilibrium concentration (mg L^{-1}), q_e the amount adsorbed (mg g^{-1}), q_m is q_e for complete monolayer adsorption capacity (mg g^{-1}), and K_a is the equilibrium adsorption constant (L mg^{-1}) and all the fits have R^2 values higher than 0.99.

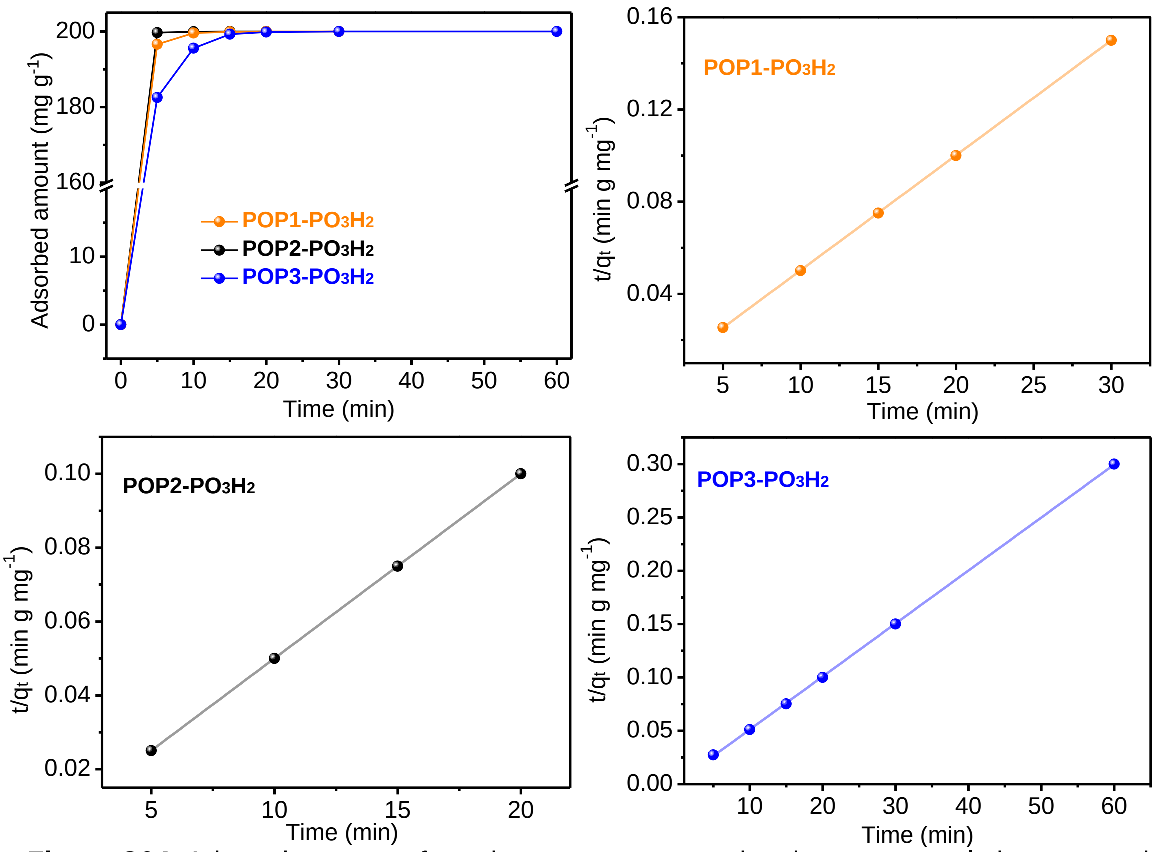

Figure S24. Adsorption curve of uranium versus contact time in aqueous solution over various adsorbents and the corresponding pseudo-second-order kinetic plot for the adsorption. The adsorption kinetic process was well-fitted with the pseudo second-order kinetic model expressed as:

$$\frac{t}{q_t} = \frac{1}{k_2 q_e^2} + \frac{t}{q_e}$$

where k_2 (g mg^{-1} min^{-1}) is the pseudo second-order rate constant of adsorption, q_t (mg g^{-1}) is the amount of uranium species adsorbed at time t (min), and q_e (mg g^{-1}) is the amount of uranium species and all the fits have R^2 values higher than 0.99. The value of the adsorption rate constant k_2 was determined to be 0.0724, 0.595, and 0.0182 g mg^{-1} min^{-1} for POP1-PO_3H_2, POP2-PO_3H_2, and POP3-PO_3H_2, respectively, indicating that POP2-PO_3H_2 exhibits a higher adsorption rate in relation to POP1-PO_3H_2 and POP3-PO_3H_2.

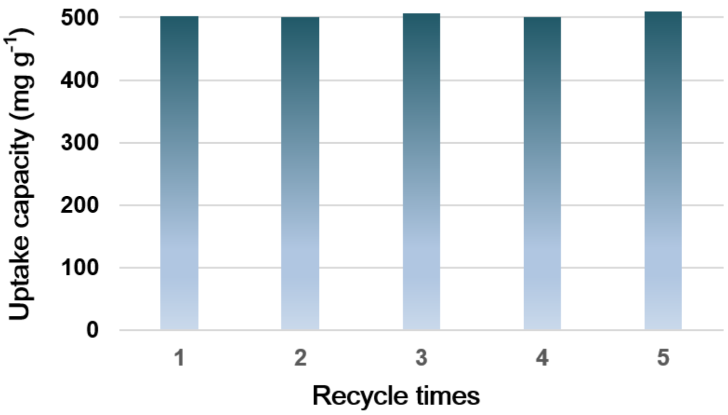

Fiigure S25. Uranium uptake capacity of POP2-PO_3H_2 as a function of the cycle number.

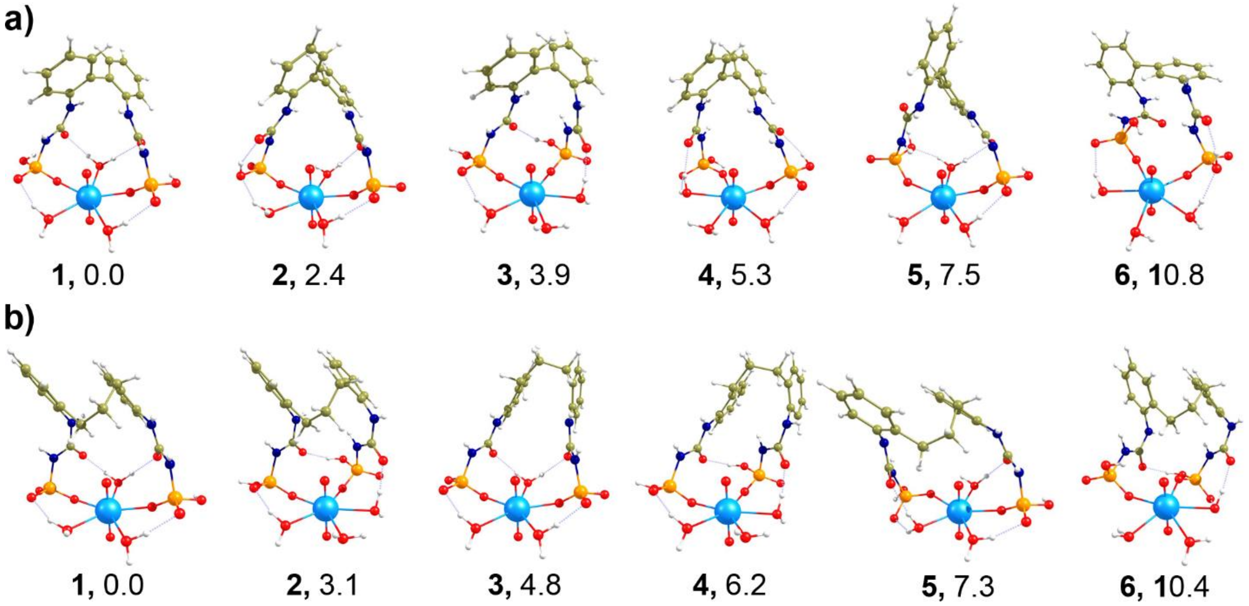

Figure S26. Structures and relative Gibbs free energies (kcal/mol) of uranyl complexes with phosphorylurea functional groups linked together through a) one C—C bond (“convergent orientation”) and b) four C—C bonds (“random orientation”) in aqueous solution. The electronic energies were obtained at the M06/SC/6-311++G(d,p) level of theory.

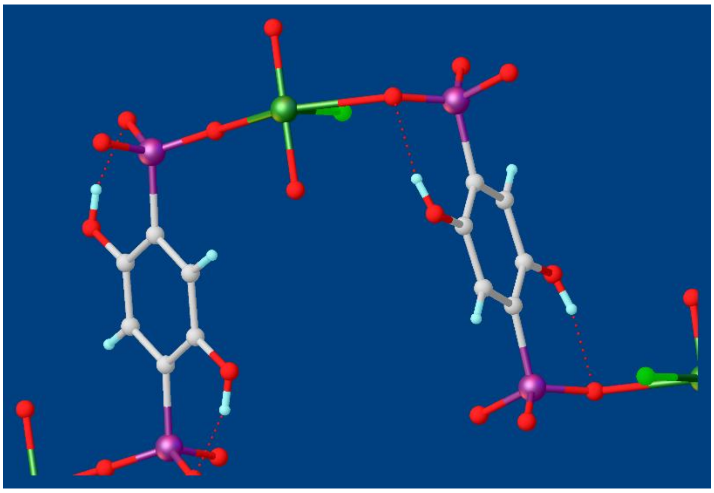

Figure 27. Cambridge Structural Database (CSD) showing exclusively monodentate phosphoryl binding for 1:1 uranyl complexes as examplified by CCDC:NOSHIO. Color legend: P, violet; O, red; C, grey; H, cyan; U, olive.

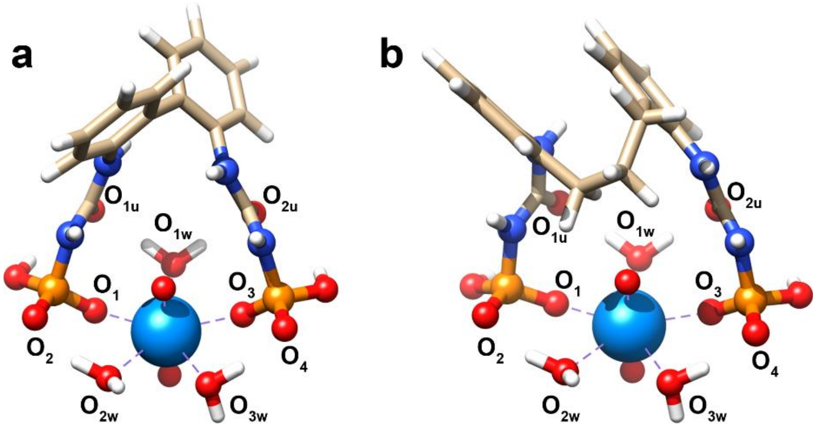

Figure S28. Optimized complex structure at the M06/SC/6-311++G(d,p) level. Color legend: P, orange; O, red; N, blue; C, beige; H, white; U, cyan.

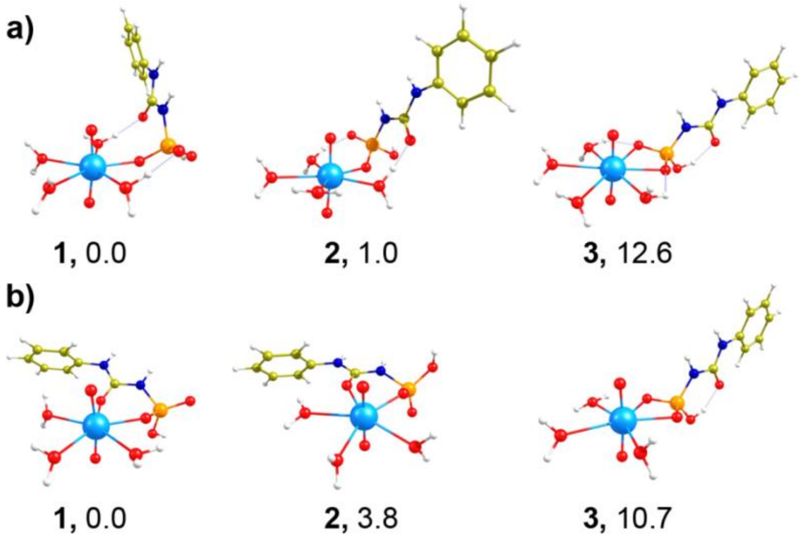

Figure S29. Structures and relative Gibbs free energies (kcal/mol) of 1:1 uranyl complexes with phosphorylurea functional group displacing a) one and b) two water molecules in the equatorial plane of the uranyl ion. DFT calculations at the M06/SC/6-311++G(d,p) level of theory were performed to elucidate the optimal coordination modes and geometries of 1:1 uranyl complexes with mono-deprotonated phosphorylurea ligand. The most stable structures of the complexes are shown above. Consistent with previous single-crystal X-ray diffraction data for phosphoryl functional groups, our calculations show that the ligand binds the uranyl cation in a monodentate fashion, while bidentate coordination mode involving two phorphoryl oxygen atoms was found to be 12.6 kcal mol^{-1} less energetically stable (**a**). Given the presence of urea oxygen donor atom in phosphorylurea functionality, the ligand can in principle form chelate complexes with uranyl (**b**) by displacing two equatorial water molecules. Our stability constant calculations indicate that the formation of such 1:1 uranyl chelate complex (log β = 6.4) is more thermodynamically favorable compared to the monodentate binding motif (log β = 3.9). Therefore, the ligand binds uranyl through phosphoryl and urea oxygens in 1:1 complex. However, when the phosphorylurea groups are joined together to afford the formation of 2:1 ligand:uranyl complexes, monodentate uranyl binding was found to be more thermodynamically stable than the corresponding chelate binding mode (Figure S28). This can be possibly explained by the enhanced stabilization of the monodentate binding in 2:1 complex due to the increased hydrogen bonding interactions between phosphorylurea groups and inner-sphere water molecules. In addition, two phosphorylurea functionalities in the linked form are constrained and seemingly cannot reorient to adopt the most favorable chelate binding configuration as compared to the free ligand.

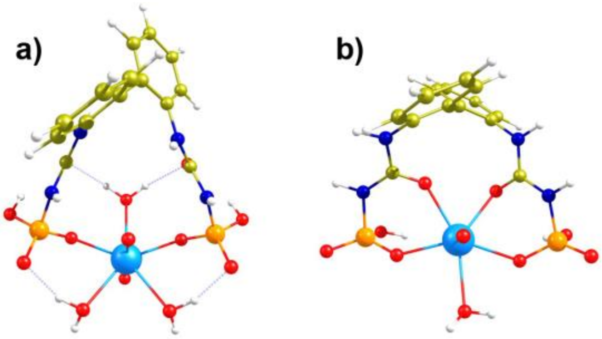

Figure S30. Comparison of calculated stability constants (log *β*) for a) monodentate and b) chelate binding motifs of 2:1 uranyl complexes.

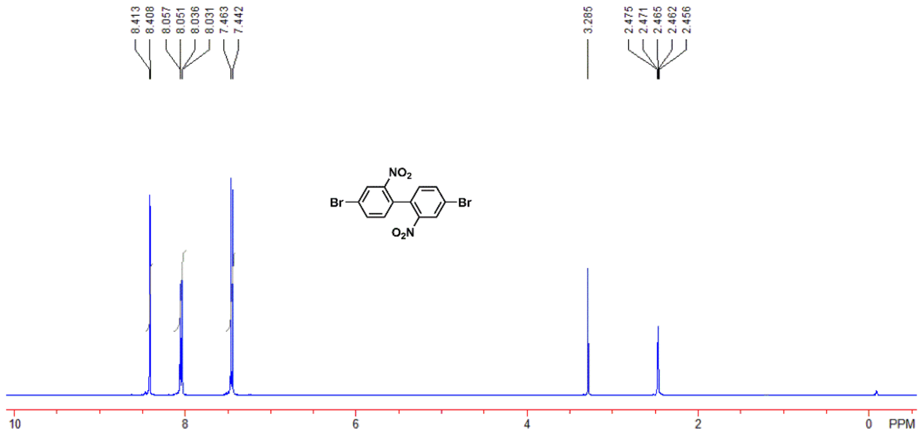
8.413
8.408
8.057
8.051
8.036
8.031
7.463
7.442
3.285
2.475
2.471
2.465
2.462
2.456
NO_2
Br
Br
O_2N
10
8
6
4
2
0
PPM

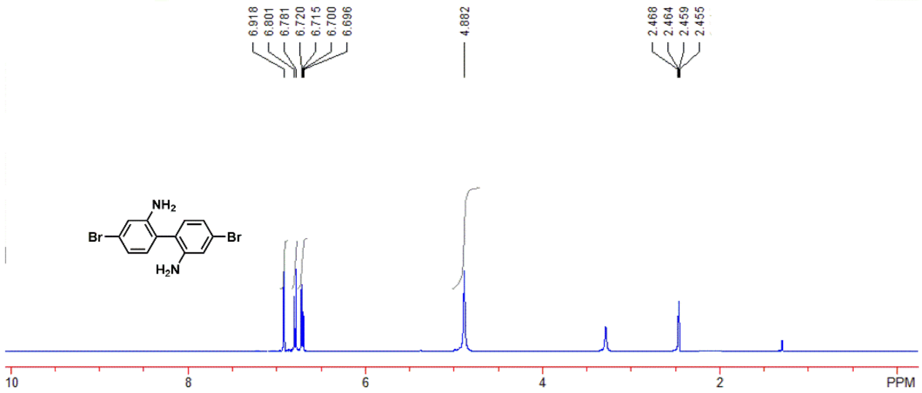
6.918
6.801
6.781
6.720
6.715
6.700
6.696
4.882
2.468
2.464
2.459
2.455
NH_2
Br
Br
H_2N
10
8
6
4
2
PPM

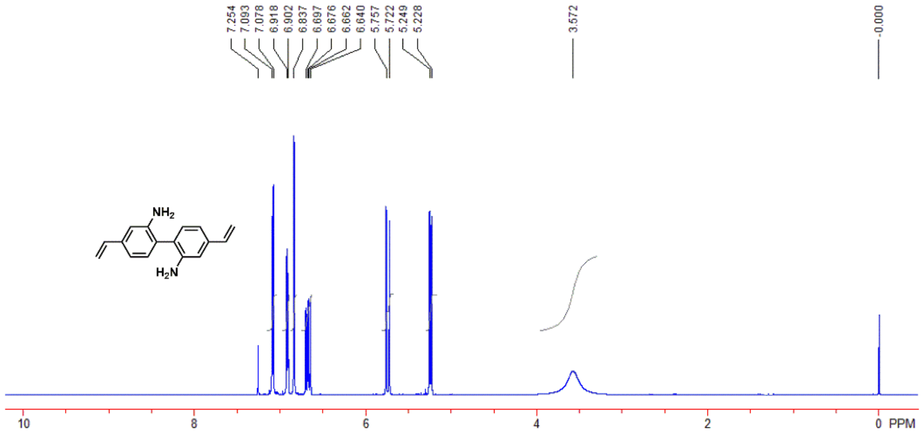
7.254
7.093
7.078
6.918
6.902
6.837
6.697
6.676
6.662
6.640
5.757
5.722
5.249
5.228
3.572
-0.000
NH_2
H_2N
10
8
6
4
2
0
PPM

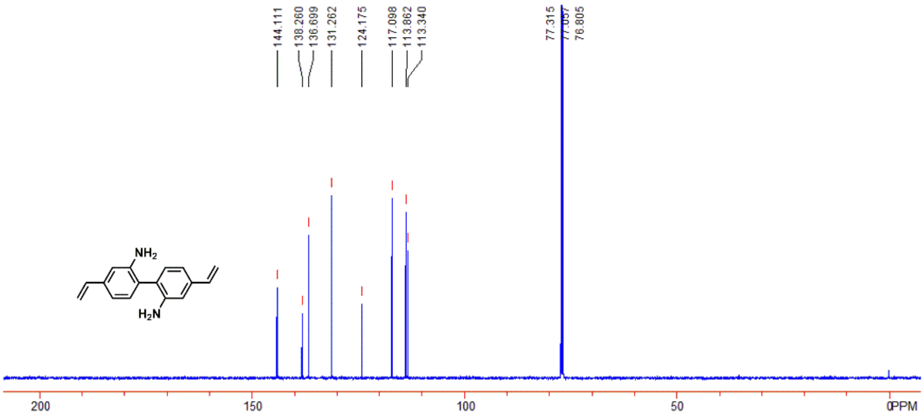
144.111
138.260
136.699
131.262
124.175
117.098
113.862
113.340
77.315
76.805
NH_2
H_2N
200
150
100
50
0PPM

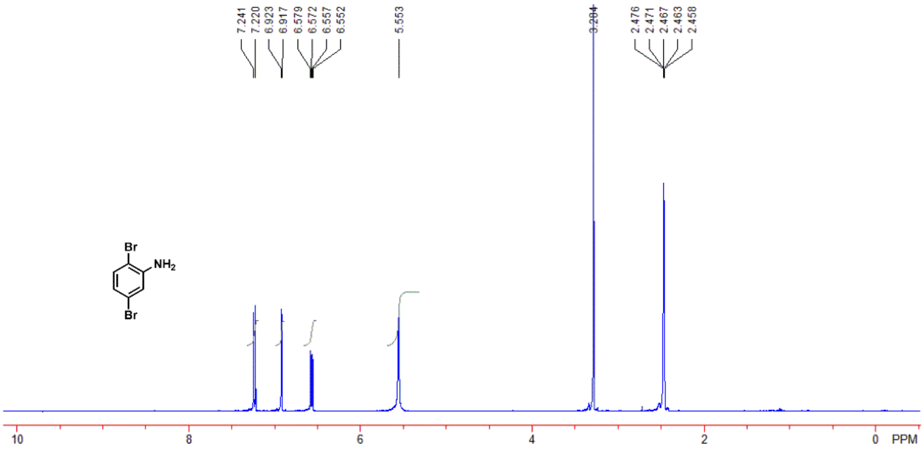
Br
NH2
Br
7.241
7.220
6.923
6.917
6.579
6.572
6.557
6.552
5.553
3.284
2.476
2.471
2.467
2.463
2.458
10 8 6 4 2 0 PPM

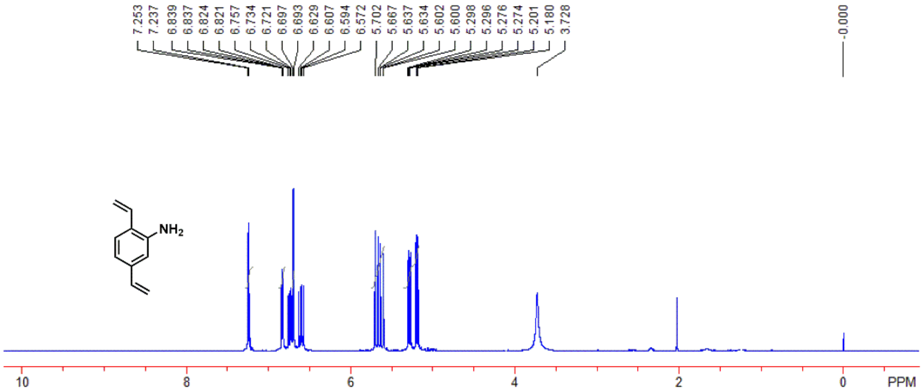
NH2
7.253
7.237
6.839
6.837
6.824
6.821
6.757
6.734
6.721
6.697
6.693
6.629
6.607
6.594
6.572
5.702
5.667
5.637
5.634
5.602
5.600
5.298
5.296
5.276
5.274
5.201
5.180
3.728
-0.000
10 8 6 4 2 0 PPM

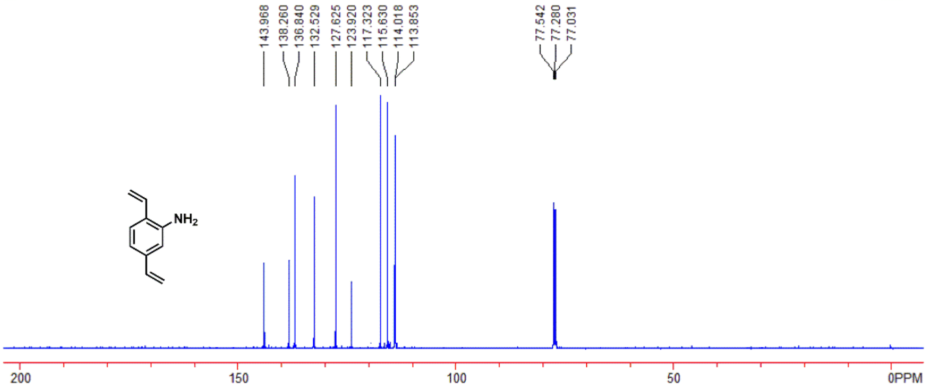
NH2
143.968
138.260
136.840
132.529
127.625
123.920
117.323
115.630
114.018
113.853
77.542
77.280
77.031
200 150 100 50 0PPM

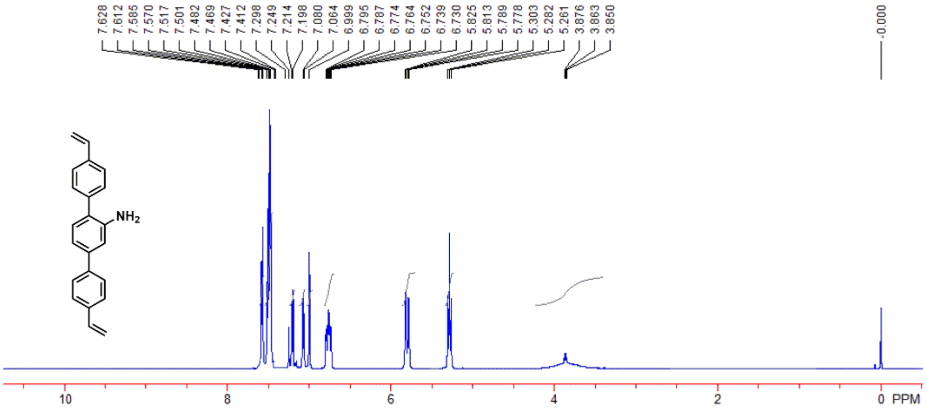
NH2
7.628
7.612
7.585
7.570
7.517
7.501
7.482
7.469
7.427
7.412
7.298
7.249
7.214
7.198
7.080
7.064
6.999
6.795
6.787
6.774
6.764
6.752
6.739
6.730
5.825
5.813
5.789
5.778
5.303
5.282
5.261
3.876
3.863
3.850
-0.000
10 8 6 4 2 0 PPM

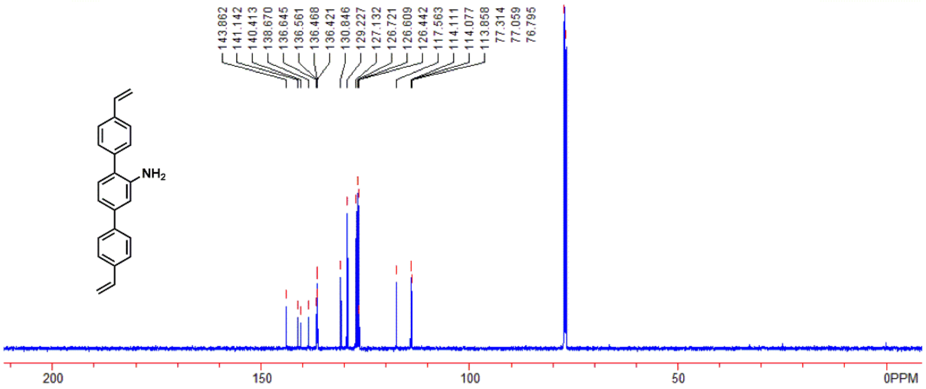

Figure S31. Liquid NMR spectra of various compounds.

References:

[1]. M. J. et al. Frisch, Gaussian 16, revision B.01; Gaussian, Inc.
[2]. Y. Zhao, D. G. Truhlar, *Theor. Chem. Acc.* **2008**, *120*, 215-241.
[3]. M. Dolg, H. Stoll, H. Preuss, R. M. Pitzer, *J. Phys. Chem*. **1993**, *97*, 5852.
[4]. A. D. Becke, J*. Chem. Phys.* **1993**, *98*, 5648.
[5]. R. F. Ribeiro, A. V. Marenich, C. J. Cramer, D. G. Truhlar, *J. Phys. Chem. B* **2011**, *115*, 14556.
[6]. A. V. Marenich, C. J. Cramer, D. G. Truhlar, *J. Phys. Chem. B* **2009**, *113*, 6378.
[7]. S. Vukovic, B. P. Hay, V. S. Bryantsev, *Inorg. Chem*. **2015**, *54*, 3995.
[8]. A. P. Ladshaw, A. S. Ivanov, S. Das, V. S. Bryantsev, C. Tsouris, S. Yiacoumi, *ACS Appl. Mater. Interfaces* **2018**, *10*, 12580.
[9]. J. P. Foster, F. Weinhold, Natural hybrid orbitals. *J. Am. Chem. Soc.* **1980**, *102*, 7211.
[10]. E. D. Glendening, C. R. Landis, F. Weinhold, *J. Comput. Chem*. **2013**, *34*, 1429.
